# Dynamic transition of the blood-brain barrier in the development of non-small cell lung cancer brain metastases

**DOI:** 10.18632/oncotarget.27274

**Published:** 2019-10-29

**Authors:** Gozde Uzunalli, Alexandra M. Dieterly, Chinyere M. Kemet, Hsin-Yi Weng, Arvin H. Soepriatna, Craig J. Goergen, Aparna B. Shinde, Michael K. Wendt, L. Tiffany Lyle

**Affiliations:** ^1^Department of Comparative Pathobiology, Purdue University, West Lafayette, IN, USA; ^2^Weldon School of Biomedical Engineering, Purdue University, West Lafayette, IN, USA; ^3^Center for Cancer Research, Purdue University, West Lafayette, IN, USA; ^4^Department of Medicinal Chemistry and Molecular Pharmacology, Purdue University, West Lafayette, IN, USA; ^5^Center for Comparative Translational Research, Purdue University, West Lafayette, IN, USA

**Keywords:** blood-brain barrier, blood-tumor barrier, non-small cell lung cancer, brain metastasis, aquaporin-4

## Abstract

Invasion of the brain by non-small cell lung cancer (NSCLC) results in a shift of the blood-brain barrier (BBB) to the insufficiently characterized blood-tumor barrier (BTB). Effective drug delivery through the BTB is one of the greatest therapeutic obstacles in treating brain metastases. Using an experimental model, we defined key changes within the BTB and the BBB in the brain around the tumor (BAT) region over time. Brain-seeking NSCLC cells were delivered into the circulation of athymic-nude mice via intracardiac injection and developing brain metastases were evaluated over six-weeks. Components of the BBB and BTB were analyzed using immunofluorescence microscopy and compared using a mixed model of regression. Our results demonstrate a dynamic time-dependent BTB phenotype. Capillaries of the BAT and BTB were dilated with increased CD31 expression compared to controls. Expression of collagen IV, a pan-basement membrane component, was significantly decreased in the BTB compared to the BBB. There was also a significant increase in the desmin-positive pericyte subpopulation in the BTB compared to the BBB. The most striking changes were identified in astrocyte water channels with a 12.18-fold (*p* < 0.001) decrease in aquaporin-4 in the BTB; the BAT was unchanged. Analysis of NSCLC brain metastases from patient samples similarly demonstrated dilated capillaries and loss of both collagen IV and aquaporin-4. These data provide a comprehensive analysis of the BTB in NSCLC brain metastasis. Astrocytic endfeet, pericytes, and the basement membrane are potential therapeutic targets to improve efficacy of chemotherapeutic delivery into NSCLC brain metastases.

## INTRODUCTION

In the United States, lung cancer is the leading cause of cancer-related deaths with a 19% five-year survival rate. In 2019, 142,670 lung cancer patients are expected to die of lung cancer in the United States [1]. Non-small cell lung cancer (NSCLC) is the most common form of lung cancer worldwide [2–4] and often metastasizes to bone, liver, and brain [5]. Ten percent of patients are diagnosed with brain metastases at the time of primary diagnosis, and 30–50% of patients are diagnosed with brain metastases over the course of the disease. Survival times range between 2–7 months and are dependent upon patient and tumor characteristics [5–7]. NSCLC brain metastases patients receive multimodal therapies including whole brain radiotherapy, stereotactic radiosurgery, surgical resection, immunotherapy and chemotherapy [7, 8]. However, due to the rapid progression of metastatic lesions, these treatment modalities are often palliative. In some cases, NSCLC brain metastases shrink with chemotherapy; unfortunately, these metastases typically recur and are resistant to additional treatment [9, 10].

The presence of the blood-brain barrier (BBB) limits the chemotherapeutic efficacy in NSCLC brain metastases. The BBB is the most restrictive vascular barrier in the body, and it is the primary site of extravasation following the invasion of neoplastic cells into the brain. It is composed of continuous endothelial cells with distinct tight junctions and efflux transporters to remove metabolic waste, basement membranes, multiple subpopulations of pericytes, and astrocyte endfeet [11]. Under normal conditions, the BBB is selectively permeable and protects the brain from circulating drugs, toxins, and pathogens. The BBB plays a critical role in controlling the exchange of molecules between the neuroparenchyma, vasculature, and cerebrospinal fluid spaces [11]. Brain metastases cause a dynamic shift of the BBB to the blood-tumor barrier (BTB). While the BTB has pathologic changes and associated increased permeability, it is not permeable enough to facilitate the delivery of large molecules [12].

We hypothesize that the growth of NSCLC brain metastases leads to time-dependent pathologic alterations in the BTB. Herein, brain metastases were formed following intracardiac injection of NSCLC cells, and novel BTB pathology was observed over six weeks. These changes were corroborated in human brain metastases specimens. Our data details the first comprehensive analysis of the transition of the BBB to the BTB in NSCLC brain metastases over time. The described BTB characteristics can serve as a foundation for improved drug delivery with an impact on patient survival and quality of life.

## RESULTS

### Brain metastases

**Figure 1:**
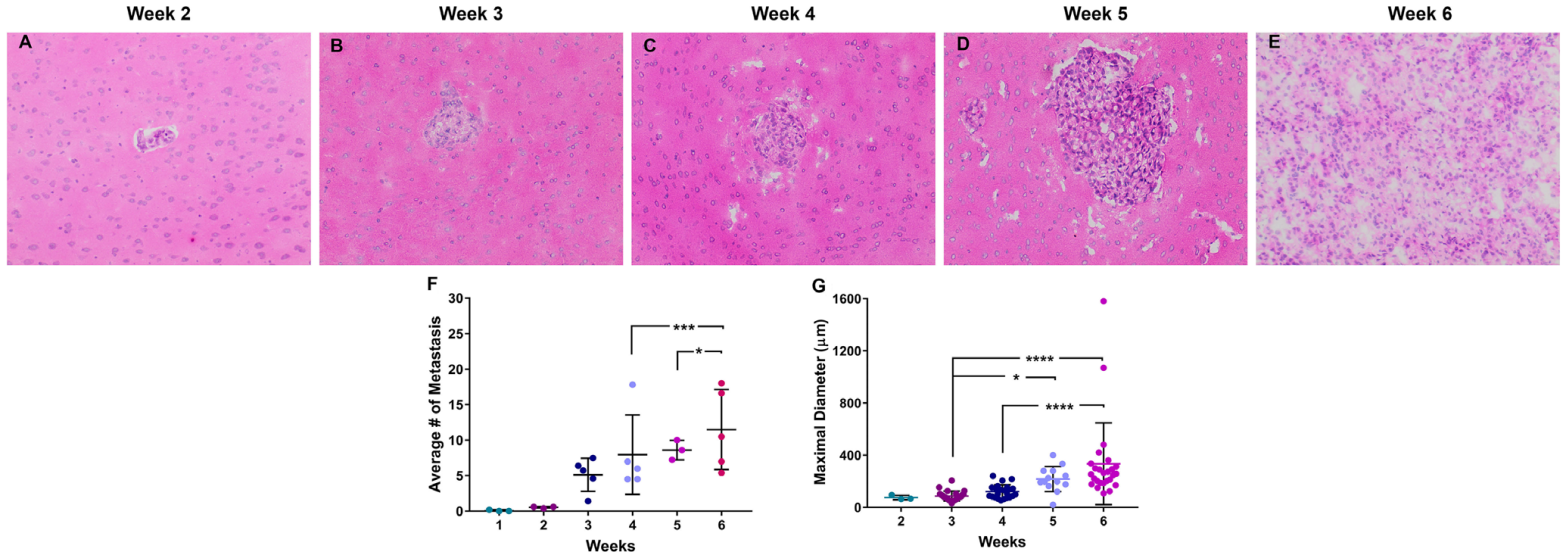
Histopathology of brain metastases of NSCLC. Representative images of NSCLC brain metastasis 2–6 weeks following intracardiac injection of A549-Br NSCLC tumor cells (**A–E**). These metastases were roughly spherical and composed of pleomorphic epithelial cells with rare necrosis and infrequent mitotic figures. The number of metastasic lesions (**F**) and diameter (**G**) of the lesions increased over a 6-week time period. All images were acquired at 100× total magnification. Error bars demonstrate standard deviation. Statistical significance was set at *p* < 0.05 (^*^ *p* < 0.05; ^**^ *p* < 0.01; ^***^ *p* < 0.001; ^****^ *p* < 0.0001).

Brain metastases were identified in three stages, early-stage at 0–2 weeks post-injection, mid-stage at 3–4 weeks post-injection, and late-stage at 5–6 weeks post-injection. Neoplastic cells were identified within one week of cellular colonization and were visualized using human mitochondria antibody (Supplementary Figure 2). In the early-stage, micrometastases (*n* = 8) were identified after two weeks of cellular colonization and were 64.6–95.3 µm in diameter. Mid-stage metastases (*n* = 286) measured between 50.9–216.9 µm, and late-stage metastases (*n* = 357) measured 20.6–1070.6 µm in diameter (Figure 1). Overall, brain metastases were roughly spherical and composed of sheets of pleomorphic neoplastic epithelial cells with abundant pale eosinophilic cytoplasm and a single nucleus. Peritumoral edema was identified around late-stage metastases, necrosis and hemorrhage were absent. One to two mitotic figures were identified in ten 40× high power fields (FN22 mm).

### Endothelial cells

**Figure 2:**
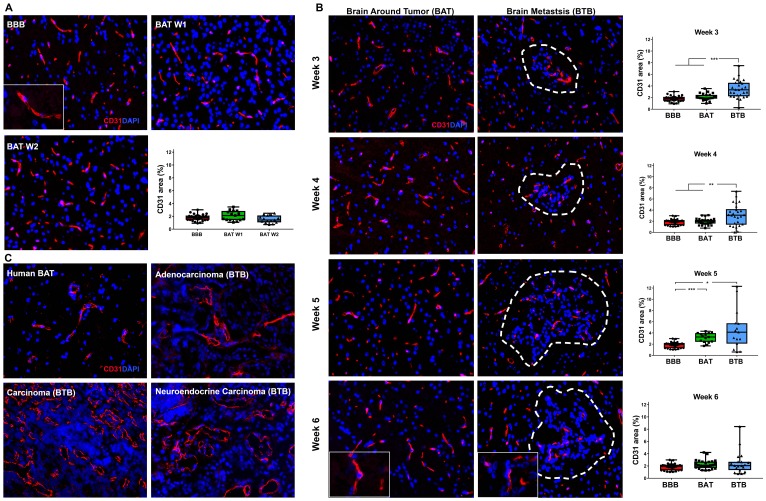
Increased expression of CD31 in brain metastases of NSCLC. Representative immunofluorescence microscopy images of capillaries (red) and early-stage (**A**), mid-, and late-stage (**B**) metastases in experimental NSCLC brain metastases and human adenocarcinoma, carcinoma and neuroendocrine carcinoma specimens (**C**). All images were acquired at 200× total magnification. Tumor margins are highlighted with a white dashed line. Within the box and whisker plot, the black line represents the mean of the data collected, box boundaries represent the 25th and 75th percentile, and error bars extend to the minimum and maximum data points. Each data point within the BAT and BBB groups represent a single image from a single animal. Each data point within the BTB group represents a single metastasis from a single animal. The level of significance was set at *p* < 0.05 (^*^ *p* < 0.05; ^**^ *p* < 0.01; ^***^ *p* < 0.001). (A) Diffuse CD31 (red) expression within BBB (*n* = 5) and BAT (*n* = 3) capillaries; nuclei were identified in blue. Quantitative analysis revealed no difference in CD31 expression the BBB and BAT at weeks 1 and week 2. (B) An increase in CD31 expression was seen as NSCLC brain metastases developed over six weeks; the most striking change was seen at 5 weeks BBB (BBB mean = 1.85, BAT mean = 3.13 BTB mean = 4.64). Tumor capillaries were densely arranged with diffuse expression of CD31 (red) compared to the BAT and BBB. (C) Vasculature of human NSCLC brain metastases was dense and tortuous with diffuse and abundant CD31 expression compared to that of the BAT.

Variation in immunofluorescence expression of the endothelial cell protein, CD31, was identified in the BTB compared to the non-tumor bearing brain around the tumor (BAT) and the BBB of control brains. Within brain sections, capillary endothelial cells were highlighted by diffuse cytoplasmic expression of CD31 (Figure 2). There was a striking increase in CD31 expression in the BTB compared to the BBB within mid and late-stage metastases (Figure 2B, Supplementary Figure 3). Within mid-stage metastases, there was an increase in CD31 expression, up to 1.90-fold, compared to the BBB (Figure 2B). CD31 expression in late-stage metastases was elevated to 2.51-fold at 5-weeks post-injection compared to the BBB; however, CD31 expression six-weeks post-injection was 1.36-fold compared to the BBB (Figure 2B). Similar to the BTB, a 1.48-fold increase in CD31 expression was identified at 5-weeks post-injection in the BAT compared to the BBB. Altogether, there was an increase in CD31 expression within both the BTB and BAT in NSCLC brain metastases (Supplementary Figure 3).

CD31 expression was evaluated in human NSCLC brain metastases specimens. In sections of NSCLC brain metastases, the vasculature of the BTB was moderately thickened and distended with increased CD31 expression compared to the BAT (Figure 2C). Increased CD31 expression in experimental and human brain metastases specimens support the transition of the BBB to the BTB in NSCLC.

### Tight junctions

Tight junctions are intercellular components of endothelial cells that are essential in controlling paracellular permeability of the intact BBB [13]. Claudins-3, 5, and 11 are tight junction proteins that have the highest impact on BBB permeability, with claudin-5 demonstrating the highest propensity for controlling paracellular permeability [14]. Distinct claudin-5 expression patterns were identified in the BBB and BTB. In the BBB, claudin-5 expression consisted of a delicate linear intercellular pattern whereas in the BTB claudin-5 protein was clumped (Supplementary Figure 4B), multifocally distributed and arranged in a roughly linear pattern. In early-stage brain metastases (week 1), a significant 1.41-fold increase in claudin-5 expression was present in the BAT compared to the BBB (Supplementary Figure 4A). Over time, there was a significant linear increase in claudin-5 expression in the BAT in mid and late-stage metastases (Supplementary Figure 5, Supplementary Table 4). Claudin-5 expression was elevated with a 1.53–1.59-fold change in the BTB in mid-stage metastases compared to the BBB (Supplementary Figure 4B).

In human NSCLC brain metastases specimens, there was a loss of claudin-5 expression in the BTB; however, in the BAT, expression was linear to threadlike, traversing between endothelial cells (Supplementary Figure 4C).

**Figure 3:**
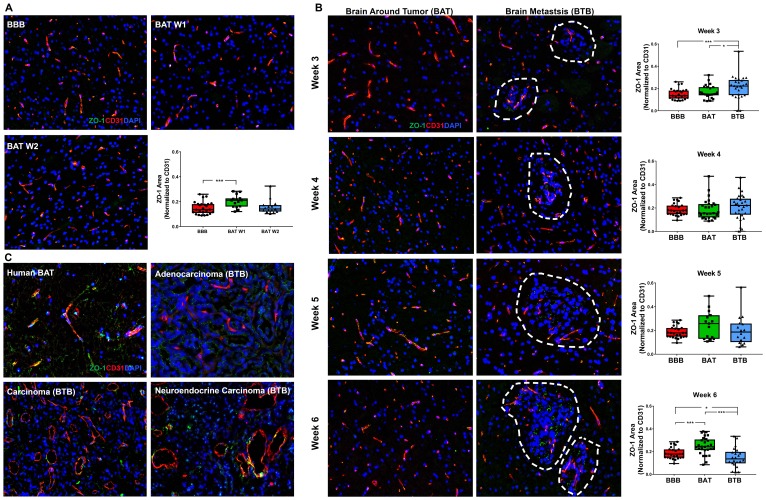
Altered ZO-1 expression in NSCLC brain metastases. Representative immunofluorescence microscopy images of ZO-1 (green) costained with CD31 (red) and DAPI (blue) within early-stage (**A**), mid- and late-stage (**B**) metastases in experimental NSCLC brain metastases and human adenocarcinoma, carcinoma and neuroendocrine carcinoma specimens (**C**). All images were acquired at 200× total magnification. Tumor margins are highlighted with a white dashed line. Within the box and whisker plot, the black line represents the mean of the data collected, box boundaries represent the 25th and 75th percentile, and error bars extend to the minimum and maximum data points. Each data point within the BAT and BBB groups represent a single image from a single animal. Each data point within the BTB group represents a single metastasis from a single animal. The level of significance was set at *p* < 0.05 (^*^ *p* < 0.05; ^**^ *p* < 0.01; ^***^ *p* < 0.001). (A) There was a significant increase in ZO-1 expression at one-week post-ICI (*n* = 3) compared to the BBB (*n* = 5). (B) Within the mid-stage metastasis, there was a significant increase in ZO-1 expression in the BTB (*n* = 5) compared to the BAT (*n* = 5) and BBB (*n* = 5). At the 6-week time point, increased ZO-1 expression was identified in BAT (*n* = 5) compared to the BBB (*n* = 5). (C) ZO-1 expression was lost in human NSCLC brain metastases capillaries.

The tight junction adapter protein, zona-occludens-1 (ZO-1), is critical for maintaining endothelial cell barriers throughout the body, including the BBB [13]. ZO-1 is known to interact closely with transmembrane proteins, including claudins, junctional adhesion molecules, occludin, and cingulin [15, 16]. We hypothesized that following the formation of brain metastases of NSCLC, there would be a loss of ZO-1 protein in the BTB compared to the BBB. In the BBB, ZO-1 maintained a threadlike pattern closely associated with endothelial cells (Figure 3A). There was a moderate increase in ZO-1 expression within the BAT at 1- and 3-weeks post-injection, compared to the BBB; this pattern was similar to the claudin-5 expression over the same time period (Supplementary Figure 4 and Supplementary Figure 5). Mid and late-stage brain metastases demonstrated widely variable expression of ZO-1 in the BAT; these trends were not significant (Figure 3B). Changes in ZO-1 expression in the BTB compared to the BBB were subtle, but began as a modest 1.42-fold (*p* < 0.001) increase at 3-weeks post-injection. Over time, there was a significant decrease in expression in ZO-1 from 3 to 6-weeks within the BTB (*p* < 0.001) (Supplementary Table 4, Supplementary Figure 6). These changes suggest that loss of ZO-1 after six-weeks of cellular colonization in the brain is a major contributor to BTB pathology. In human brain metastases specimens, there was a loss of ZO-1 expression in the BTB compared to the BAT (Figure 3C). The expression pattern in the BAT was clumped and often lacked linear continuity.

The time-dependent expression patterns of claudin-5 and ZO-1 within the BTB suggest that while the proteins are present, they may not be biologically functional due to the wide variation in expression patterns.

### Basement membrane

**Figure 4:**
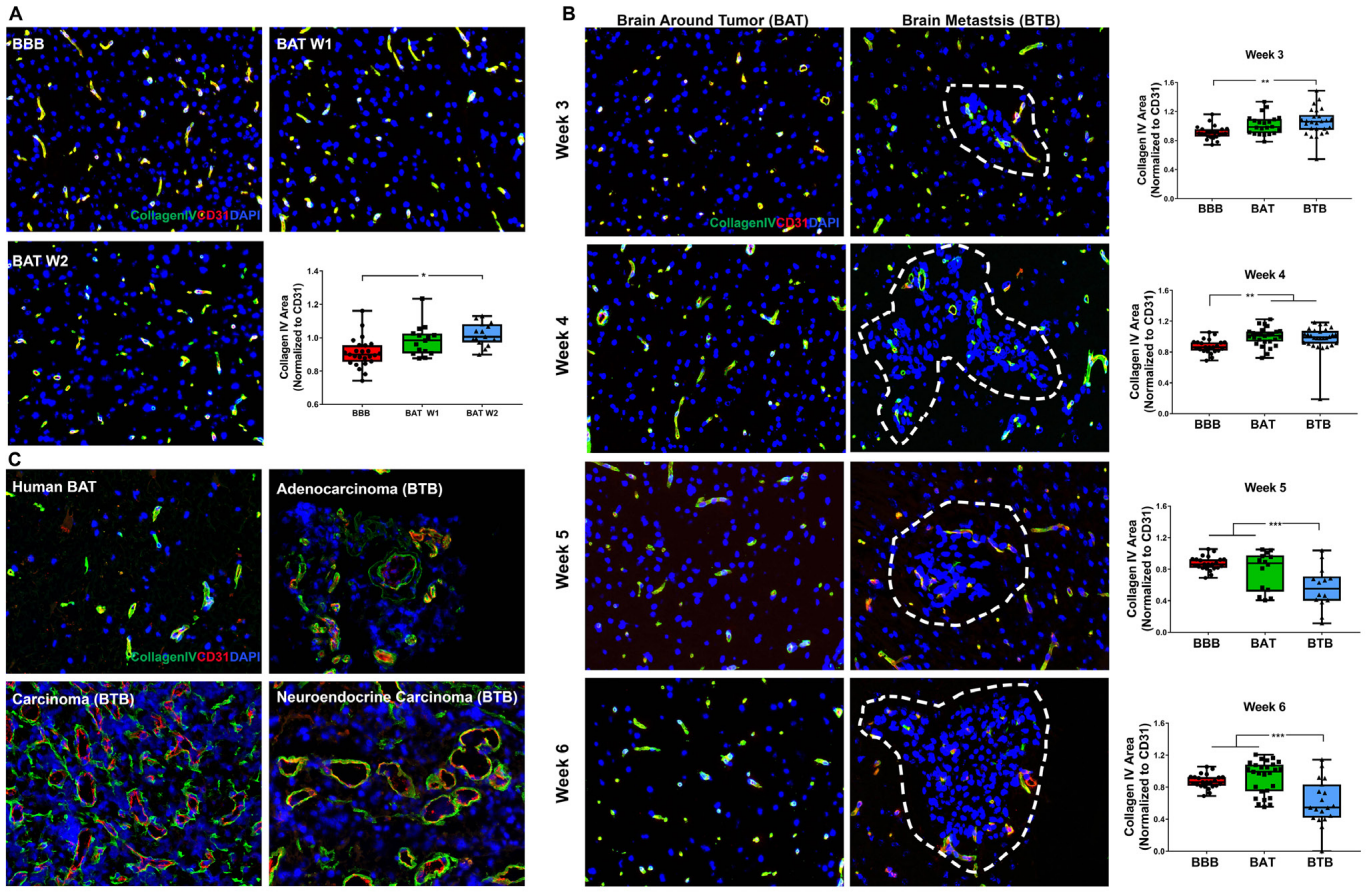
Collagen IV protein expression was diminished within late-stage NSCLC brain metastasis. Representative immunofluorescence microscopy images of the basement membrane (green) costained with CD31 (red) and DAPI (blue) in early-stage (**A**), mid-, and late-stage (**B**) metastases in experimental NSCLC brain metastases and human adenocarcinoma, carcinoma and neuroendocrine carcinoma specimens (**C**). All images were acquired at 200× **total** magnification. Tumor margins are highlighted with a white dashed line. Within the box and whisker plot, the black line represents the mean of the data collected, box boundaries represent the 25th and 75th percentile, and error bars extend to the minimum and maximum data points. Each data point within the BAT and BBB groups represent a single image from a single animal. Each data point within the BTB group represents a single metastasis from a single animal. The level of significance was set at *p* < 0.05 (^*^ *p* < 0.05; ^**^ *p* < 0.01; ^***^ *p* < 0.001). (A) In early-stage metastases, there was a significant increase in COLIV expression within the BAT (week 1 and 2, *n* = 3) at week 2 compared to the BBB (*n* = 5). (B) Mid-stage metastasis (*n* = 5) demonstrated a minor increase in COLIV expression within the BTB compared to the BBB and BAT (*n* = 5). However, there was a significant loss of COLIV protein within the BTB of late-stage metastases (week 5 *n* = 3, week 6 *n* = 5) compared to the BBB and BAT. (C) Capillaries within human NSCLC brain metastases specimens had a discontinuous expression of COLIV compared to the diffuse and continuous expression within the BAT.

The basement membrane is a three-dimensional, 20–200 nm, interconnected network of collagens, laminins, nidogens, and heparan sulfate proteoglycans. These components form a barrier between endothelial cells, glia, and neurons [17, 18]. We evaluated the basement membrane microscopically using the pan-basement membrane protein, collagen IV (COLIV), for early, mid and late-stage metastases (Figure 4A–4B). Within the BTB of late-stage metastases, there was a modest loss of COLIV at five (1.55-fold; *p* < 0.001) and six (1.42-fold; *p* = 0.008) weeks post-injection compared to the BBB (Figure 4B). A significant loss of collagen IV expression in the BTB was present from three to six weeks of cellular colonization, but this trend was not observed within the BAT (Supplementary Figure 7, Supplementary Table 4). In human brain metastases specimens, capillaries of the BTB had discontinuous expression of COLIV and mild perivascular edema. By contrast, capillaries of the BAT exhibited diffuse expression of COLIV (Figure 4C).

Laminin-α2 (LAMA2) is a component of the astrocytic basement membrane and serves as a receptor ligand. BAT and BTB expression patterns were similar 3–5 weeks post-injection (Supplementary Figure 8). At 5-weeks post-intracardiac injection, a significant increase in BAT and BTB expression was identified compared to the BBB, with a 1.61-fold (*p* < .001) and 1.56-fold (*p* = 0.007) increase, respectively (Supplementary Figure 8B). There was a significant increase in LAMA2 in late-stage NSCLC brain metastases (Supplementary Table 4 and Supplementary Figure 9). The greatest expression of LAMA2 within the BAT was at 6-weeks post-injection with a 1.78-fold (*p* < 0.001) increase compared to the BBB. In human brain metastases specimens, LAMA2 was similarly decreased in the BTB compared to the BAT (Supplementary Figure 8C). Expression patterns of COLIV and LAMA2 provide a pathway for the development of molecular targets for chemotherapeutic delivery.

### Pericytes

**Figure 5:**
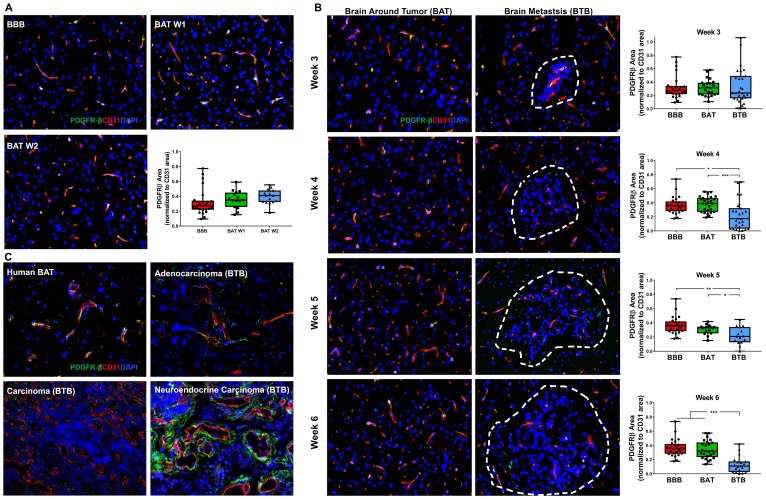
Loss of PDGFR-β protein expression in NSCLC brain metastases. Representative immunofluorescence microscopy images of PDGFR-β-positive pericytes (green) costained with CD31 (red) and DAPI (blue) within early-stage (**A**), mid- and late-stage (**B**) metastases in experimental NSCLC brain metastases and human adenocarcinoma, carcinoma and neuroendocrine carcinoma specimens (**C**). All images were acquired at 200× total magnification. Tumor margins are highlighted with a white dashed line. Within the box and whisker plot, the black line represents the mean of the data collected, box boundaries represent the 25th and 75th percentile, and error bars extend to the minimum and maximum data points. Each data point within the BAT and BBB groups represent a single image from a single animal. Each data point within the BTB group represents a single metastasis from a single animal. The level of significance was set at *p* < 0.05 (^*^ *p* < 0.05; ^**^ *p* < 0.01; ^***^ *p* < 0.001). (A) Multifocally throughout the BBB and BAT, PDGFR-β-positive pericytes were costained with CD31-positive endothelial cells (yellow). No significant change in PDGFR-β-positive pericytes was present within the BAT of early-stage metastases (week 1 and 2, *n* = 3), compared to the BBB (*n* = 5). (B) There was a loss of expression of PDGFR-β-positive pericytes within the BTB compared to the BAT. PDGFR-β protein expression within the BTB decreased at mid (*n* = 5) and late-stage (week 5 *n* = 3, week 6 *n* = 5) metastases compared to the BAT and BBB. (C) Loss of PDGFR-β was observed in the human NSCLC carcinoma specimen compared to the BAT; however, aberrant PDGFR-β-positive pericytes were identified within the adenocarcinoma and neuroendocrine carcinoma brain metastasis specimens.

Platelet-derived growth factor receptor-β (PDGFR-β), a pan-pericyte protein, modulates signaling pathways to maintain BBB integrity, homeostasis, and vascular tone. Although there was no significant change within early-stage metastases (Figure 5A), PDGFR-β-positive pericytes were diminished in the BTB of mid and late-stage NSCLC brain metastases. At 4-weeks post-injection, there was a 1.66-fold decrease in PDGFR-β expression in the BTB compared to the BBB (*p* = 0.027). The loss of PDGFR-β expression continued through late-stage metastases, where at 6-weeks post-injection, there was a 2.78-fold decrease in expression compared to the BBB (*p* < 0.001) (Figure 5B). Minimal variation in PDGFR-β expression in the BAT and BTB from 1–6 weeks post-intracardiac injection was seen (Supplementary Figure 10, Supplementary Table 4). These data demonstrate a loss of PDGFR-β-positive pericytes within the developing BTB and retention of PDGFR-β expression in the BAT over time. In human brain metastases, PDGFR-β was closely associated with endothelial cells of the BAT, but there was a loss of PDGFR-β expression in the carcinoma specimen. Variable PDGFR-β expression patterns were identified in the adenocarcinoma and neuroendocrine carcinoma specimens (Figure 5C).

CD13 is an alanyl aminopeptidase, which is a cell surface marker in the brain pericyte vasculature identified in resting pericytes [19, 20]. CD13 plays a role in tumor invasion, angiogenesis, cellular proliferation, migration, and differentiation [21]. There was no significant difference between the BBB and BAT in early-stage metastases (Supplementary Figure 11). Overall, there was an increase in CD13 expression in the BTB and BAT in NSCLC brain metastases; this trend was most prominent at week 4 (BTB 2.42-fold (*p* = 0.001); BAT 1.78-fold (*p* < 0.001)). CD13 expression patterns were similar in the BAT and BTB 3–6-weeks post-injection (Supplementary Figure 12 and Supplementary Figure 13).

**Figure 6:**
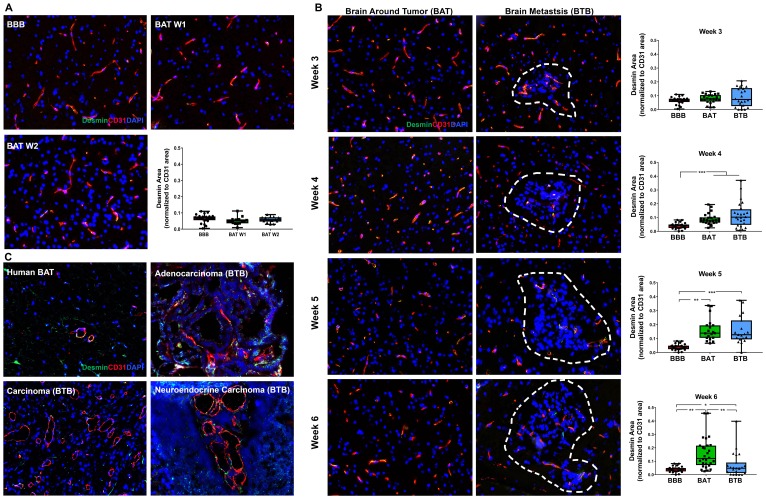
Expression of desmin-positive pericytes correlated with the development of NSCLC brain metastases. Representative immunofluorescence microscopy images of desmin (green) costained with CD31 (red) and DAPI (blue) at early-stage (**A**), mid-, and late-stage (**B**) metastases in experimental NSCLC brain metastases and human adenocarcinoma, carcinoma and neuroendocrine carcinoma specimens (**C**). All images were acquired at 200× total magnification. Tumor margins are highlighted with a white dashed line. Within the box and whisker plot, the black line represents the mean of the data collected, box boundaries represent the 25th and 75th percentile, and error bars extend to the minimum and maximum data points. Each data point within the BAT and BBB groups represent a single image from a single animal. Each data point within the BTB group represents a single metastasis from a single animal. The level of significance was set at *p* < 0.05 (^*^ *p* < 0.05; ^**^ *p* < 0.01; ^***^ *p* < 0.001). (A) There was scant, multifocal expression of desmin, co-stained with CD31 (yellow) within the BBB (*n* = 5) and BAT (week 1 and 2, *n* = 3)in early stage-metastases. (B) Moderate, multifocal expression of desmin-positive pericytes co-stained with CD31 (yellow) was identified in mid and late-stage metastases of the BAT and BTB. (A) significant increase in desmin-positive pericyte expression was identified at week 4 (*n* = 5), week 5 (*n* = 3) and week 6 (*n* = 5) within the BTB of NSCLC brain metastases. (C) Multifocal and aberrant expression of desmin was identified throughout the BAT and BTB of human adenocarcinoma, carcinoma, and neuroendocrine NSCLC brain metastases specimens.

Desmin is a type III intermediate filament protein, abundant in cardiac, skeletal, and smooth muscle cells, in addition to a subtype of contractile pericytes [22]. Although there was no change in early-stage metastases (Figure 6A), a significant increase in desmin expression was identified in the BTB and BAT 4–6 weeks post-injection compared to the BBB (Supplementary Figure 14). The greatest shift in desmin-positive pericytes was identified in late-stage metastases at 5-weeks post-injection with a 3.95-fold (*p* < 0.001) increase in the BTB and a 4.10-fold increase (*p* = 0.003) in the BAT compared to the BBB (Figure 6B). Overall, there was a time-dependent and significant shift in desmin-positive pericyte expression in NSCLC brain metastases (Supplementary Table 4). Desmin expression in human metastases specimens was spurious and inconclusive (Figure 6C).

### Astrocyte endfeet

**Figure 7:**
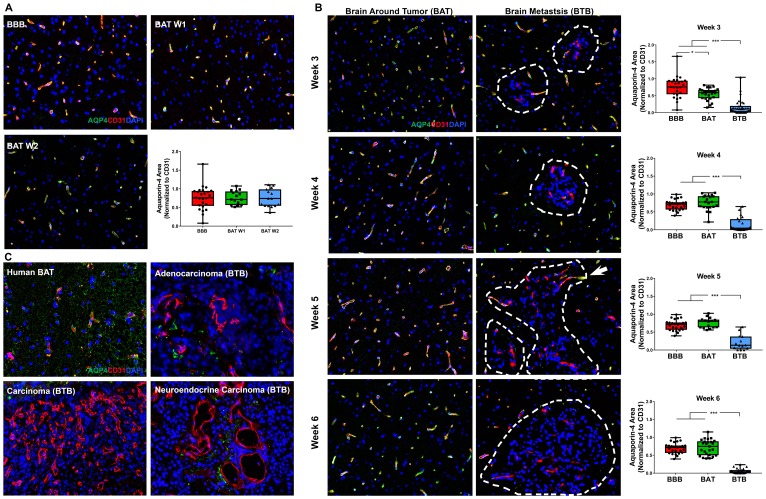
Catastrophic loss of the astrocytic endfeet water channel protein aquaporin-4 in NSCLC brain metastases. Representative immunofluorescence microscopy images of aquaporin-4 (green) costained with CD31 (red) and DAPI (blue) in early-stage (**A**), mid-, and late-stage (**B**) metastases in experimental NSCLC brain metastases and human adenocarcinoma, carcinoma and neuroendocrine carcinoma specimens (**C**). All images were acquired at 200× total magnification. Tumor margins are highlighted with a white dashed line. Within the box and whisker plot, the black line represents the mean of the data collected, box boundaries represent the 25th and 75th percentile, and error bars extend to the minimum and maximum data points. Each data point within the BAT and BBB groups represent a single image from a single animal. Each data point within the BTB group represents a single metastasis from a single animal. The level of significance was set at *p* < 0.05 (^*^ *p* < 0.05; ^**^ *p* < 0.01; ^***^ *p* < 0.001). (A) Within the BAT and BBB, there was multifocal to diffuse co-expression of AQP-4 and CD31 (yellow) within early-stage metastases. (B) Capillaries of the BTB lacked expression of AQP4, while capillaries of the BAT had multifocal to diffuse expression of AQP4 and CD31 (yellow). Few capillaries at the brain-tumor interface maintained co-expression of AQP4; this was most evident at week 5 (white arrow). NSCLC brain metastases exhibited a time-dependent loss of aquaporin-4 protein at mid (*n* = 5) and late-stage (week 5 *n* = 3, week 6 *n* = 5) metastases. (C) Within human NSCLC brain metastases specimens, capillaries of the BAT maintained AQP4 expression. However, loss of AQP4 expression was identified within the metastatic lesions of adenocarcinoma, carcinoma and neuroendocrine carcinoma.

Aquaporin-4 (AQP4) is a water channel within astrocytic endfeet that is critical for the delivery of nutrients to surrounding neurons [23]. There was no change in AQP4 expression in early-stage brain metastases (Figure 7A). As NSCLC brain metastases developed, a significant loss of AQP4 expression within the BAT was identified in week 3 (Supplementary Figure 15). Loss of AQP4 was identified in both the BTB and BAT 3-weeks post-injection with a 4.65-fold (*p* < 0.001) decrease and 1.37-fold decrease (*p* = 0.001), respectively (Figure 7B). Of all functional components of the BTB, the most striking BTB pathology was identified in AQP4. Within the BTB, a loss of AQP4 expression persisted throughout the development of mid and late-stage metastases, and this linear trend was significant (Supplementary Figure 15, Supplementary Table 4). At 6-weeks, there was a pronounced 12.18-fold decrease (*p* < 0.001) in AQP4 expression within the BTB compared to the BBB. These findings were correlated with human brain metastases specimens, which demonstrated a loss of expression of AQP4 in adenocarcinoma, carcinoma, and neuroendocrine carcinoma specimens compared to the BAT (Figure 7C). These data demonstrate loss of polarization of the astrocytic endfeet, and therefore, loss of functioning water channels.

### Reactive astrogliosis

**Figure 8:**
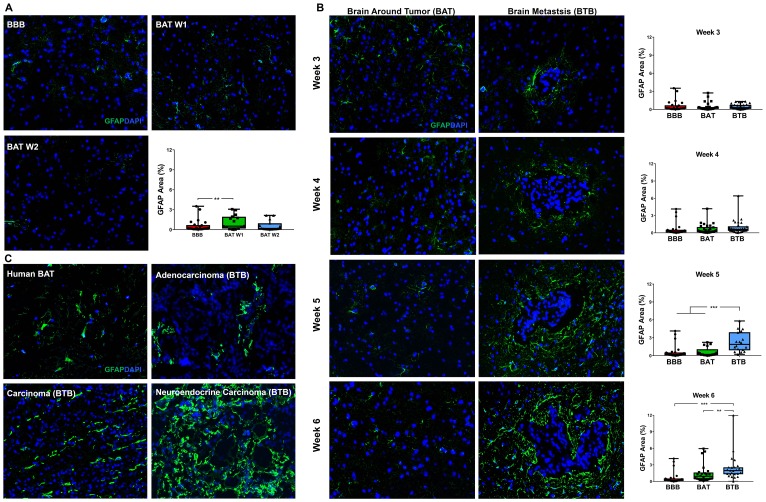
Reactive astrocytosis in NSCLC brain metastases. Representative immunofluorescence microscopy images of GFAP (green) costained with DAPI (blue) in early-stage (**A**), mid- and late-stage (**B**) metastases in experimental NSCLC brain metastases and human adenocarcinoma, carcinoma and neuroendocrine carcinoma specimens (**C**). All images were acquired at 200× total magnification. Tumor margins are highlighted with a white dashed line. Within the box and whisker plot, the black line represents the mean of the data collected, box boundaries represent the 25th and 75th percentile, and error bars extend to the minimum and maximum data points. Each data point within the BAT and BTB groups represent a single image from a single animal. Each data point within the BTB group represents a single metastasis from a single animal. The level of significance was set at *p* < 0.05 (^*^ *p* < 0.05; ^**^ *p* < 0.01; ^***^ *p* < 0.001). (A) Rare reactive astrocytes were identified within the BAT within early stage metastases compared to the BBB. After week 1, there was a slight increased GFAP protein expression in BAT (week 1 and 2, *n* = 3), compared to the BBB (*n* = 5). (B) A proliferation of hypertrophic reactive astrocytes was identified in mid-stage metastases but was most prominent in late-stage metastases. While mid-stage (*n* = 5) reactive astrocytosis remains similar around the metastatic lesions, there was a significant increased GFAP expression at late-stage metastases (week 5 *n* = 3, week 6 *n* = 5). (C) Hypertrophic, reactive, GFAP-positive astrocytes were identified within the BAT and most prominent within and around brain metastases.

In the normal brain, there was rare expression of the reactive astrocyte protein, glial fibrillary acidic protein (GFAP) (Figure 8A). Within the experimental model, there was a 3.46–3.68-fold (*p* < 0.001) increase in GFAP expression within late-stage metastases (Figure 8B). Our data demonstrate an ascending trend in GFAP expression tightly encircling metastases beginning 4-weeks post-injection (Supplementary Figure 16). In late stage metastases, there was a 3.71-fold (*p* < 0.001) and 1.64-fold (*p* = 0.009) increase in GFAP expression in the BTB compared to the BAT (Figure 8B). Within human NSCLC brain metastases specimens, a small population of GFAP-positive reactive astrocytes were identified within the BAT. Proliferation of reactive astrocytes were identified throughout the adenocarcinoma, carcinoma, and neuroendocrine carcinoma specimens (Figure 8C). Our data demonstrate a correlation between tumor cell growth and proliferation of reactive astrocytes, and this change may interfere with chemotherapeutic efficacy.

## DISCUSSION

The BBB protects the brain from the invasion of harmful substances and permits the passive or transcellular movement of nutrients and small molecules. This unique vascular barrier is composed of endothelial cells with distinct tight junctions, a parenchymal and endothelial basement membrane, pericytes embedded within the basement membrane, and polarized astrocyte endfeet [11]. Together these components create an impenetrable structure that controls the movement of small molecules that traverse the neuroparenchyma [24, 25].

We demonstrated the dynamic transition of the BBB to the BTB in an experimental model of NSCLC brain metastasis. Over 6 weeks, we evaluated changes in the functional BBB components, including endothelial cells, basement membranes, pericytes, and astrocyte endfeet. We hypothesized that as tumor cells invade the brain, there is a distinct change in the morphologic characteristics of the BBB as it shifts to the BTB. Our findings provide the first comprehensive evaluation of the BBB, BAT, and BTB in NSCLC brain metastases.

Treatment of brain metastases is a challenge in the clinic due to the formation of the BTB and heterogeneity of metastatic tumor cells [26, 27]. The vasculature within and around brain metastases is more permeable than the vasculature of the normal brain in animal models and human brain metastases specimens [12, 27, 28]. The absence of vascular pathology has been described in early-stage NSCLC brain metastases, and this trend was also present in our model system [29] (Supplementary Figure 1). Vasculature within mid and late-stage NSCLC brain metastases consisted of increased CD31 expression and vascular dilation. Similarly in human brain metastases specimens, dilated capillaries exhibited increased CD31 expression as described by Fidler *et al.* [30]. Our findings in NSCLC brain metastasis are also supported by recent studies that demonstrate elevated CD31 expression in cervical carcinoma brain metastases and experimental models of brain metastases of breast cancer [27, 31, 32].

Tight junctions are known to be a critical and differentiating feature of continuous brain capillaries in contrast to fenestrated or discontinuous capillaries present in other parenchymal organs. Claudins are linked to tight junction adaptor proteins, including zona occludens-1 (ZO-1), within the endothelial cell cytoplasm [15, 16]. In the experimental model of NSCLC brain metastasis, we identified a unique claudin-5 expression pattern and trend. Claudin-5 expression was increased in mid-stage and late-stage metastases, with a clumped and thickened expression pattern often focal and randomly located along the capillary endothelial cells, in contrast to the linear threadlike claudin-5 pattern of BBB controls. As NSCLC brain metastases developed, a decrease in ZO-1 expression within the BTB was present in both our preclinical model and in human specimens. The functionality of the tight junctions and associated adapter proteins depends on the expression level as well as spatiotemporal arrangement of these complex proteins [13]. As claudin-5 expression increased over time, the pattern of expression was altered and suggestive of a lack of functionality. With this change in expression pattern, there may be a lack of cohesion for the tight junction adapter protein ZO-1, which may explain the precipitous decrease in ZO-1 expression in the BTB. A similar trend has been described in experimental models of brain metastases of breast cancer where there was a loss of ZO-1 and an increase in claudin-5 in the BTB [27]. Within NSCLC brain metastases, ZO-1 expression was identified between neoplastic epithelial cells. Intercellular and intracellular expression of ZO-1 has been described in NSCLC and has been correlated with poor prognosis in patients [33].

Aberrant localization of claudins and zona-occludens have been associated with perturbations of the BBB in other cancers, including glioma [34, 35].

The BBB consists of endothelial and astrocytic basement membranes [36]. Collagen IV [α1(IV) _2_α2(IV)] is the most abundant extracellular matrix (ECM) protein in the endothelial basement membrane and is crucial for development, maintenance, and functionality of the BBB [37]. The astrocytic basement membrane contains laminin isoforms, predominantly laminin-α2 [18]. Astrocytic laminin maintains the integrity of the BBB by modulating pericyte differentiation and maturation [25]. Degradation of collagen IV has been identified in both acute and chronic neurodegenerative diseases, most notably in ischemic stroke [38]. Similarly, we demonstrated a loss of collagen IV expression in the BTB of experimental NSCLC brain metastases. By contrast, a significant increase in astrocytic laminin-α2 was seen in the BAT; however, the expression in the BTB was variable and equivalent to the BBB after 6 weeks of colonization. In human brain metastases specimens, there was a vast difference in expression of collagen IV in the BTB compared to the BAT, characterized by the discontinuity in expression and evidence of perivascular edema separating the endothelium and the basement membrane. Interestingly in LAMA2, a loss of expression in the BTB compared to the BAT was also characterized by a discontinuous pattern of expression. These data demonstrate changes in the basement membrane that may be predictive of BBB permeability. The increase in LAMA2 in the BAT and variability in the BTB are unique compared to other brain metastases models, particularly in metastatic breast cancer. In brain metastases of breast cancer, there was a loss of LAMA2, which correlated with an increase in BTB permeability [27]. A similar pattern was reported by Yao *et al*.; they demonstrated a correlation between astrocytic laminin and tight junction expression [39]. The haphazard and globular morphology of the tight junction proteins in NSCLC brain metastases may be the result of altered basement membranes of the BTB.

Pericytes, which are divided into several heterogeneous subtypes, are present on the abluminal surface of the endothelium [40]. Although endothelial cells and pericytes are separated by the basement membrane, the two cells are in direct contact [25]. Pericytes contribute to the integrity and stability of the BBB, proliferation of endothelial cells, and deposition of ECM [41].

PDGFR-β is a pan-pericyte protein and a receptor for endothelial cell-derived PDGF-B. Absence of PDGFR-β or PDGF-B is associated with embryonic lethality and vascular dysfunction [42]. PDGF-B truncating mutations result in impaired BBB function with increased permeability [24]. We identified a loss of PDGFR-β within the BTB of mid- and late-stage experimental NSCLC brain metastases and human NSCLC brain metastases specimens. Metastatic colonization of breast cancer cells in the brain gives rise to a similar phenotype with loss of PDGFR-β-positive pericytes [27]. These findings demonstrate the importance of PDGFR-β-positive pericytes in BBB structure and function.

CD13-positive pericytes play a role in vascular tone and regulating the inflammatory response [24]. We identified an increase in CD13 expression in mid-stage metastases within the BTB and BAT. Although these changes were not evaluated in human specimens due to challenges in antibody optimization, Matteo *et al.* demonstrated elevated CD13 expression in capillaries within several human tumor specimens [21].

An increase in contractile, desmin-positive pericytes were identified in the BAT and BTB after 3–5 weeks of cellular colonization. However, in human brain metastases, desmin expression was inconclusive in adenocarcinoma and carcinoma brain metastases. This finding may be due to the chronicity of the lesion in human patients, compared to the experimental model. In mid-stage metastases, there was a steady decrease in PDGFR-β expression, with a simultaneous increase in CD13 and desmin-positive pericyte expression. This variation in expression is likely associated with differences in pericyte subtypes and biological functionality of these cells. The question remains if there is a proliferation of pericytes or differentiation from the resting to the contractile phenotype. Moreover, this data is in direct contrast to pericyte trends identified in brain metastases of breast cancer, which demonstrated a loss of PDGFR-β-positive pericytes, a loss in CD13-positive pericytes, and increase in desmin-positive pericytes [27]. The difference in expression patterns within brain metastases of NSCLC compared to breast cancer further demonstrate the heterogeneity that exists between two metastatic cancers colonizing the neuroparenchyma.

Polarization of astrocyte endfeet around the BBB basement membrane is critical for maintaining BBB integrity and restricting vascular permeability [43]. Aquaporin-4 water channels are a primary component in these polarized astrocyte endfeet. In the BTB there was a dramatic loss (3.35–12.18 fold) of AQP-4 compared to the BBB in mid and late-stage metastases. In the BAT, expression levels were similar to that of the BBB for all weeks, except week 3 where the formation of micrometastases was apparent. Vessels that were at the tumor interface exhibited a precipitous loss of AQP-4 within the tumor parenchyma. A loss of AQP-4 was also present within all human NSCLC brain metastases specimens. Loss of AQP-4 has been reported in Parkinson’s disease and associated with inflammation and in experimental models of breast cancer brain metastases [27, 44]. These data suggest that aquaporin-4 and polarized endfeet are paramount in maintaining the structural integrity of the BBB.

As tumor cells colonize the brain, reactive astrocytes protect the parenchyma from further damage and further limit intratumoral chemotherapeutic delivery. The proliferation of reactive astrocytes within and surrounding the tumor parenchyma was evident throughout the experimental and human brain metastases of NSCLC in our study. There was a modest increase in reactive astrocytes in the BAT; however, this trend was not significant. In human specimens, the most striking increase in GFAP expression was within the neuroendocrine carcinoma; however, reactive astrocytes were identified throughout the BAT and the BTB of adenocarcinoma and carcinoma. Reactive and hypertrophic astrocytes have been associated with neurologic diseases, including inflammation, neurodegeneration, and cancer [45].

**Figure 9:**
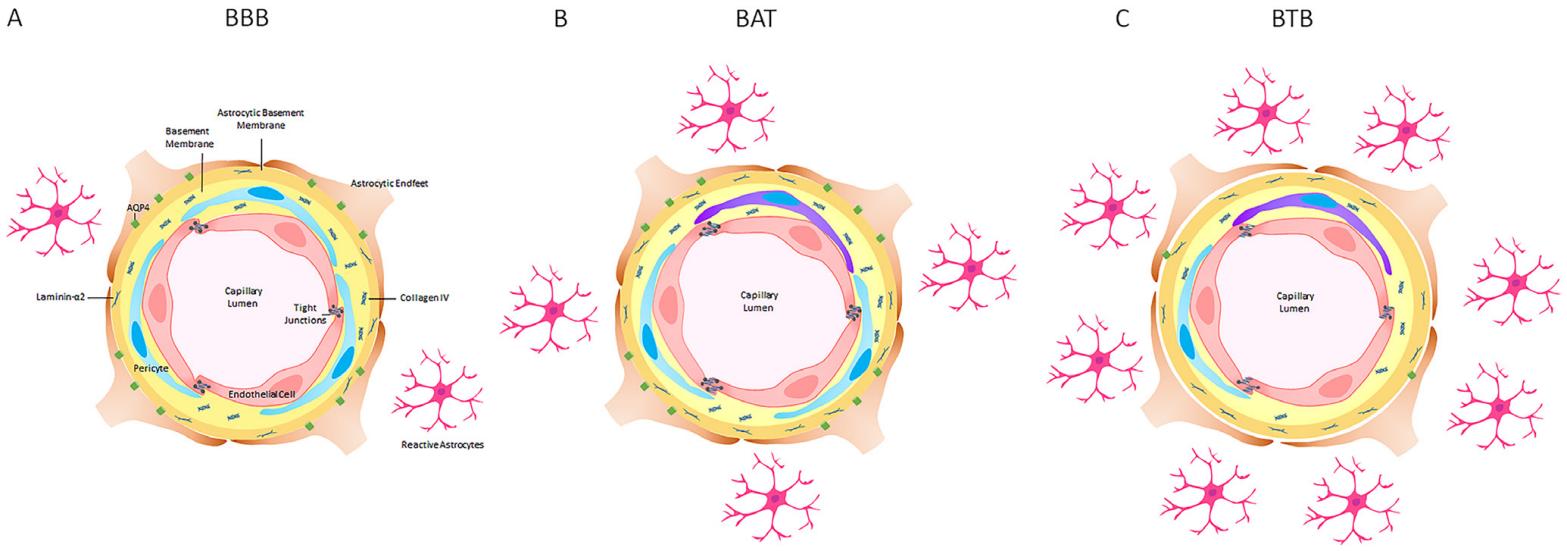
A schematic representation of the blood-brain barrier (BBB), brain around tumor region (BAT) and blood-tumor barrier (BTB) in late stage NSCLC brain metastases in an experimental model. (**A**) The BBB consists of endothelial cells (red), PDGFR-β-positive pericytes (blue) embedded in the endothelial (yellow) and astrocytic basement membranes (orange) enclosed by polarized astrocytic endfeet (brown) with aquaporin-4 water channels (green). (**B**) The tight junctions and basement membrane of the BAT were altered with an increase in claudin-5 protein, a loss of ZO-1 adapter protein and an increase in laminin-α2 protein in the astrocytic basement membrane. (**C**) In the BTB, there was a prominent loss of collagen IV in the endothelial basement membrane, PDGFR-β-positive pericytes and aquaporin-4 water channel protein. GFAP+ astrocytes (pink) and desmin+ contractile pericytes (purple) were identified throughout tumor-bearing brain sections.

This comprehensive analysis correlated BTB and BAT pathology in an experimental model of brain metastatic NSCLC and human NSCLC brain metastases specimens. Over time, BTB activity was most prominent after 5 weeks of cellular colonization with variation in expression in all functional components. Overall, the most striking changes were in the loss of aquaporin-4, loss of zona occludens-1, and loss of collagen-IV within the BTB (Figure 9A–9C), and these changes were corroborated in human brain metastases. Altogether, these data demonstrate that in the late stage metastases, the transition of the BBB to the BTB is stepwise and validated in clinical samples. A limitation of this comprehensive time course study was antibody sensitivity and formation of distant metastases, including the vertebrae [46]. A complete understanding of the physiological response is limited by the use of immunocompromised animals. This research has provided the framework to explore the heterogeneity that exists in the BTB and BAT in the development of brain metastases of NSCLC. Correlation of BTB pathology with permeability and drug delivery mechanisms are critical to build on this seminal work.

## MATERIALS AND METHODS

### Cell culture and maintenance

Brain-seeking A549 NSCLC tumor cells (A549-Br) were kindly provided by Dr. Patricia Steeg, National Cancer Institute, Bethesda, MD. A549-Br cells were cultured with 10% fetal bovine serum (Gibco-BRL, Grand Island, NY, USA), 1% penicillin/streptomycin and RPMI 1640 with 1% L-glutamine (Life Technologies; Carlsbad, CA, USA). Cells were maintained in a 37°C incubator with 5% CO_2_. A549-Br cells were independently authenticated at the American Type Culture Collection (Manassas, VA, USA).

### Lentiviral transduction

Twelve micrograms of plasmid were transfected with psPAX2 (4 µg) and pMD2.G (4 µg) into HEK293T cells using polyethyleneimine (3 µl/µg) to obtain lentiviral particles. A549-Br cells were cultured in RPMI 1640 medium and were transduced with lentiviral particles for 48 hours. Stably transduced cells were selected over 15 days in zeocin (200 µg/ml). Luciferase-expressing A549-Br cells were maintained at 85% confluency with A549-Br culture medium in a 37°C, 5% CO_2_ incubator, and the medium was replaced every 2–3 days.

### NSCLC brain metastases model

All *in vivo* experiments were approved by the Purdue University Animal Care and Use Committee. Animals were maintained with *ad libitum* access to water and feed in a 12 h−12 h light−dark cycle. NSCLC brain metastases were established using 25 g, 6-week-old Hsd:Athymic Nude-Foxn1nu mice (RRID:MGI:5652489, Envigo, Indianapolis, IN, USA). Animals were euthanized after 1 (*n* = 3), 2 (*n* = 3), 3 (*n* = 5), 4 (*n* = 5), 5 (*n* = 3), and 6 (*n* = 5) weeks of cellular colonization or with evidence of clinical demise. Nine (*n* = 5) and twelve-week-old (*n* = 5) control animals were paired with animals euthanized after 1–3 weeks or 4–6 weeks of cellular colonization.

Ultrasound-guided intracardiac injection was performed using the methods described in Dieterly *et al.* [46]. Briefly, 1x10^6^ A549-Br NSCLC tumor cells were injected after visualization of the needle inside the left ventricle (MS550D transducer, Vevo2100, FUJIFILM VisualSonics; Toronto, Ontario, Canada). One-hour post-intracardiac injection, D-luciferin (150 mg/kg, Gold Biotechnology, Olivette, MO, USA) was delivered via intraperitoneal injection (Supplementary Figure 1). Animals were imaged using a Spectral Ami Optical Imaging System and AMI View software (Spectral Instruments Imaging, Tucson, AZ, USA).

### Histopathology of brain metastases

Immediately following euthanasia, brains were removed, flash frozen in a slurry of ethanol and dry ice, and embedded in OCT. Five-micrometer brain sections were stained with hematoxylin and eosin (HE) and coverslipped using standard histology techniques in a Leica ST5010-CV5030 integrated workstation. Metastatic lesions were also confirmed by two pathologists following HE staining. Images were acquired at 100× total magnification using an Olympus BX43 microscope and analyzed with LCmicro v2.2 software.

### Immunofluorescence microscopy

Immediately after euthanasia, brains were removed and flash frozen in a slurry of ethanol and dry ice. Brains were cryosectioned in 5 µm serial sections and stored at –80°C. Brain sections containing metastatic lesions were confirmed with HE staining and adjacent sections were used for immunofluorescence assay. Immunofluorescence assays were performed with a 5-minute PBS wash at 4°C. Slides were fixed in methanol or acetone, depending on the antibody for 5 minutes. Tissues sections were blocked with 5% normal goat serum in PBS for 45 minutes, and tissue sections were incubated with antibodies for 16–18 hours at 4°C. All antibodies are listed in Supplementary Tables 1 and 2. Alexa Fluor 488 conjugated goat anti-mouse IgG, Alexa Fluor 488 conjugated goat anti-rabbit IgG or Alexa Fluor 568 conjugated goat anti-rat IgG (1:500) were used as secondary antibodies. All samples were prepared in parallel with the negative control. All samples were mounted onto glass slides using ProLong^®^ anti-fade mounting medium with DAPI (Life Technologies).

### Image analysis

Digital images were acquired using a Zeiss Axio Scope A2 (Carl Zeiss Microimaging, GmbH, Jena, Germany) at 200× magnification and exposure times for each channel were standardized for each antibody. Blood-brain barrier (BBB) proteins were analyzed using Zen Blue software (Carl Zeiss Microimaging, GmbH, Jena, Germany). The surface area of each BBB protein was measured within an immunofluorescent image for the BBB and BAT (brain around the tumor) or within a metastatic lesion for the BTB. Percent area was calculated by measuring the density of claudin-5, zona occludens-1, collagen IV, platelet-derived growth factor receptor-β, desmin, or aquaporin-4 normalized to CD31. Laminin-α2 and CD13 were not co-stained with CD31; these antibodies required different staining conditions or were harvested in the same species as CD31. Raw unsaturated images were used for all quantitative analyses.

### Statistical analysis

A mixed model of regression was used to analyze the data to account for repeated sampling. Statistical analyses were performed using Stata (Release 14. StataCorp LP. College Station, TX, USA). All graphs were developed using GraphPad Prism, version 7.0, (GraphPad Software Incorporated, La Jolla, CA, USA). Statistical significance was indicated at *p* < 0.05. Summary of the fold changes and statistics were provided in Supplementary Tables 2 and 3.

## SUPPLEMENTARY MATERIALS




