# Supplementary material for: Dynamic transition of the blood-brain barrier in the development of non-small cell lung cancer brain metastases
Source: Oncotarget. 2019 Oct 29;10(59):6334–48. doi: 10.18632/oncotarget.27274 (PMC6824867; doi:10.18632/oncotarget.27274)
Supplement: Supplementary file 1 [file oncotarget-10-6334-s001.pdf]

Dynamic transition of the blood-brain barrier in the development of non-small cell lung cancer brain metastases

SUPPLEMENTARY MATERIALS

SUPPLEMENTARY METHODS

Immunofluorescence of human NSCLC brain metastasis specimens

Snap-frozen human NSCLC brain metastasis specimens were obtained from the Indiana University Simon Cancer Center. Under the auspices of a protocol approved by the Indiana University Institutional Review Board, all patients consented for participation. Specimens with confirmed histology and diagnosis were routinely available within 90 minutes post-excision. The BTB was evaluated in NSCLC adenocarcinoma, carcinoma, and neuroendocrine carcinoma specimens. The BAT was evaluated in a section of brain harboring large cell carcinoma, but devoid of tumor cells. Tissues were embedded in OCT, cryosectioned at 5 µm thickness, and fixed with methanol or acetone. Primary antibodies used are shown in Supplementary Table 2.

Supplementary Table 1: Antibodies evaluated in an experimental model of NSCLC brain metastases

Component	Antibody	Vendor and Catalog Number	Dilution	RRID
Astrocytes	Glial Fibrillary Acidic Protein (GFAP)	Millipore (MAB360)	1:10,000	AB_11212597
Endothelial Cells	CD31	BD Pharmingen (550274)	1:500	AB_393571
Endothelial Cell Tight Junctions	Zona Occludin-1	Invitrogen (61-7300)	1:100	AB_2533938
Astrocyte Endfeet	Aquaporin 4	Millipore (AB3594)	1:7500	AB_91530
Endothelial Cell Tight Junctions	Claudin-5	Life Technologies (34-1600)	1:50	AB_86930
Basement Membrane	Collagen IV	Millipore (ab756P)	1:200	AB_2276457
Astrocytic Basement Membrane	Laminin-α2	Abcam (ab11576)	1:100	AB_298180
CD13+ Pericytes	CD13	Abcam (ab33489)	1:50	AB_726095
Desmin+ Pericytes	Desmin	Dako (M0760)	1:50	AB_2335684
Pericytes	PDGFR-β	Abcam (ab32570)	1:100	AB_777165
NSCLC tumor cells	Human mitochondria	Abcam (ab92824)	1:100	AB_10562769

Supplementary Table 2: Antibodies evaluated in human NSCLC brain metastases specimens

Component	Antibody	Vendor and Catalog Number	Dilution	RRID
Astrocytes	Glial Fibrillary Acidic Protein (GFAP)	Millipore (MAB360)	1:15,000	AB_11212597
Endothelial Cells	CD31	Invitrogen (13-0319-82)	1:50	AB_466423
Endothelial Cell Tight Junctions	Zona Occludin-1	Invitrogen (61-7300)	1:100	AB_2533938
Astrocyte Endfeet	Aquaporin 4	Millipore (AB3594)	1:7500	AB_91530
Endothelial Cell Tight Junctions	Claudin-5	Life Technologies (34-1600)	1:50	AB_86930
Basement Membrane	Collagen IV	Millipore (ab756P)	1:200	AB_2276457
Astrocytic Basement Membrane	Laminin-α2	Abcam (ab11576)	1:100	AB_298180
Desmin^{+}-Pericytes	Desmin	Abcam (Ab15200)	1:250	AB_301744
Pericytes	PDGFR-β	Abcam (ab32570)	1:50	AB_777165

Supplementary Table 3: Summary of fold changes and statistics for each analyzed immunofluorescent antibody. Summary of mean values, fold changes, and statistics for each analyzed immunofluorescent marker. (NS: Not significant). See Supplementary_Table_3

Supplementary Table 4: Summary of time-dependent statistics of each analyzed immunofluorescence antibody

BBB Component	*Group*	*Weeks*	*P values*
CD31	BAT	Week 3 vs. 5	< 0.001
		Week 4 vs. 5	< 0.001
		Week 5 vs. 6	0.008
	BTB	Week 3 vs. 6	0.009
Claudin 5	BAT	Week 1 vs. 2	0.086 (NS)
		Week 3 vs. 4	< 0.001
		Week 3 vs. 5	0.046
		Week 3 vs. 6	0.010
	BTB	Week 3 vs. 4	< 0.001
ZO-1	BAT	Week 1 vs. 2	0.043
	BTB	Week 3 vs. 6	< 0.001
		Week 4 vs. 6	0.002
Collagen IV	BTB	Week 3 vs. 5	< 0.001
		Week 3 vs. 6	< 0.001
		Week 4 vs. 5	< 0.001
		Week 4 vs. 6	< 0.001
Laminin-α2	BAT	Week 3 vs. 6	0.011
		Week 4 vs. 5	0.011
		Week 4 vs. 6	0.001
	BTB	Week 3 vs. 6	0.038
		Week 5 vs. 6	0.018
PDGFR-β	BTB	Among all weeks	0.056 (NS)
Desmin	BAT	Week 3 vs. 5	0.029
		Week 3 vs. 6	0.049
	BTB	Week 3 vs. 5	0.026
		Week 4 vs. 6	0.009
		Week 5 vs. 6	0.001
CD13	BAT	Week 3 vs. 4	0.002
Aquaporin-4	BAT	Week 3 vs. 4	0.002
		Week 3 vs. 5	< 0.001
		Week 3 vs. 6	0.023
	BTB	Week 3 vs. 6	0.002
		Week 4 vs. 6	0.005
		Week 5 vs. 6	0.006
GFAP	BAT	Week 1 vs. 2	0.001
	BTB	Week 3 vs. 4	0.006
		Week 3 vs. 5	< 0.001
		Week 3 vs. 6	< 0.001
		Week 4 vs. 5	< 0.001
		Week 4 vs. 6	0.004

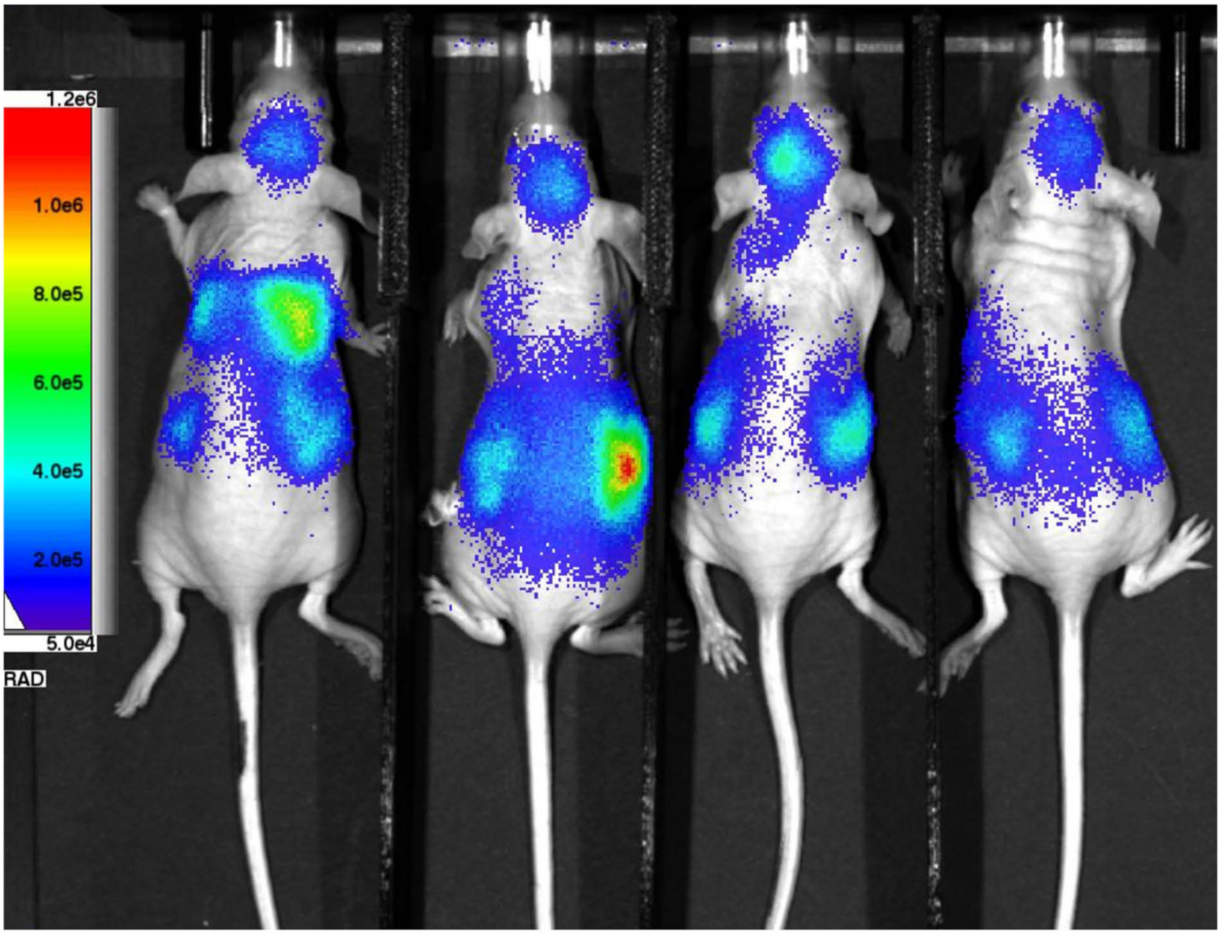

Supplementary Figure 1: Representative *in vivo* imaging of athymic nude mice following intracardiac injection of A549-Br NSCLC tumor cells. One-hour post-intracardiac injection, D-luciferin (150 mg/kg) was delivered via intraperitoneal injection. The Spectral Ami Optical Imaging system was used to confirm and monitor the metastatic growth in the brain over time.

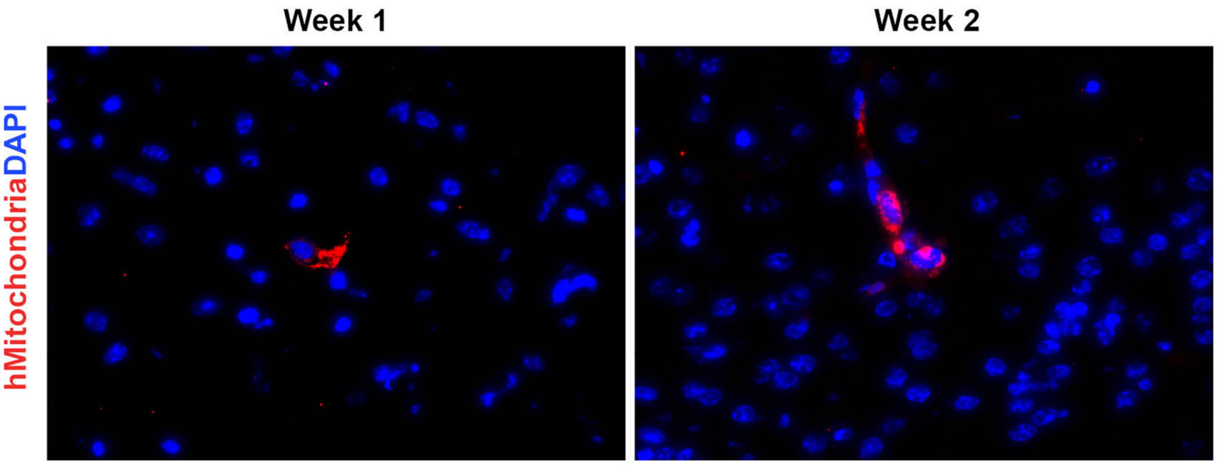

Supplementary Figure 2: Immunofluorescence image of human mitochondria-positive A549-Br cells one-week and two-weeks post-intracardiac injection. Nuclei were identified with DAPI (blue), and human mitochondria-positive tumor cells were identified in red. Images were acquired at 200X total magnification.

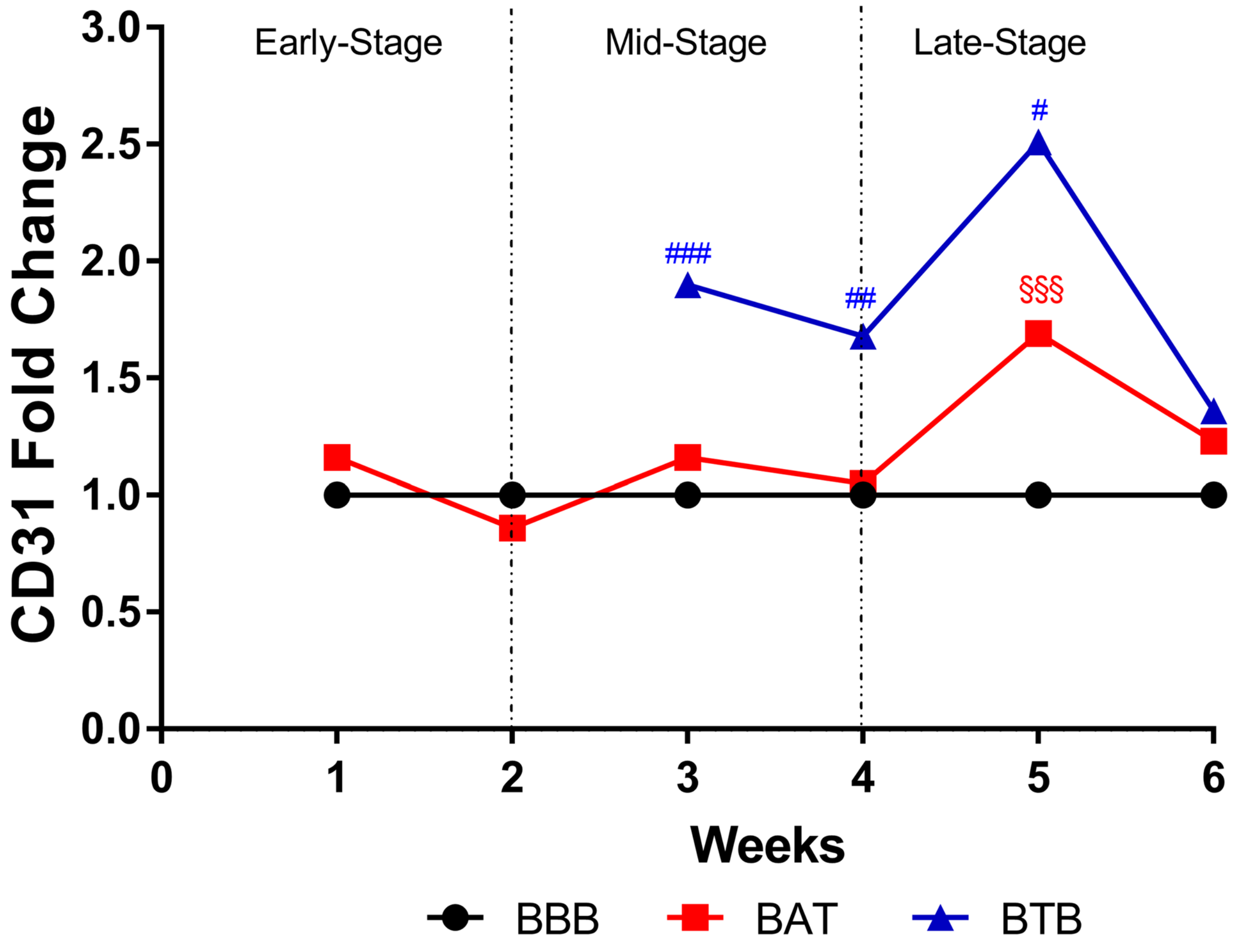

Supplementary Figure 3: Time-dependent quantitative analysis of CD31 expression within the BBB, BAT, and BTB in NSCLC brain metastases. There was a significant increase in CD31 expression within the BTB over time. The line graph demonstrates quantitative analysis of CD31 in early stage (1–2 weeks), mid-stage (3–4 weeks) and late stage (5–6 weeks) metastases. The BBB is held constant at 1.0, and each data point represents the overall fold-change within the BTB and BAT. The level of significance was set at $p < 0.05$ ($^{\#,\S}p < 0.05$; $^{\#\#,\S\S}p < 0.01$; $^{\#\#\#,\S\S\S}p < 0.001$).

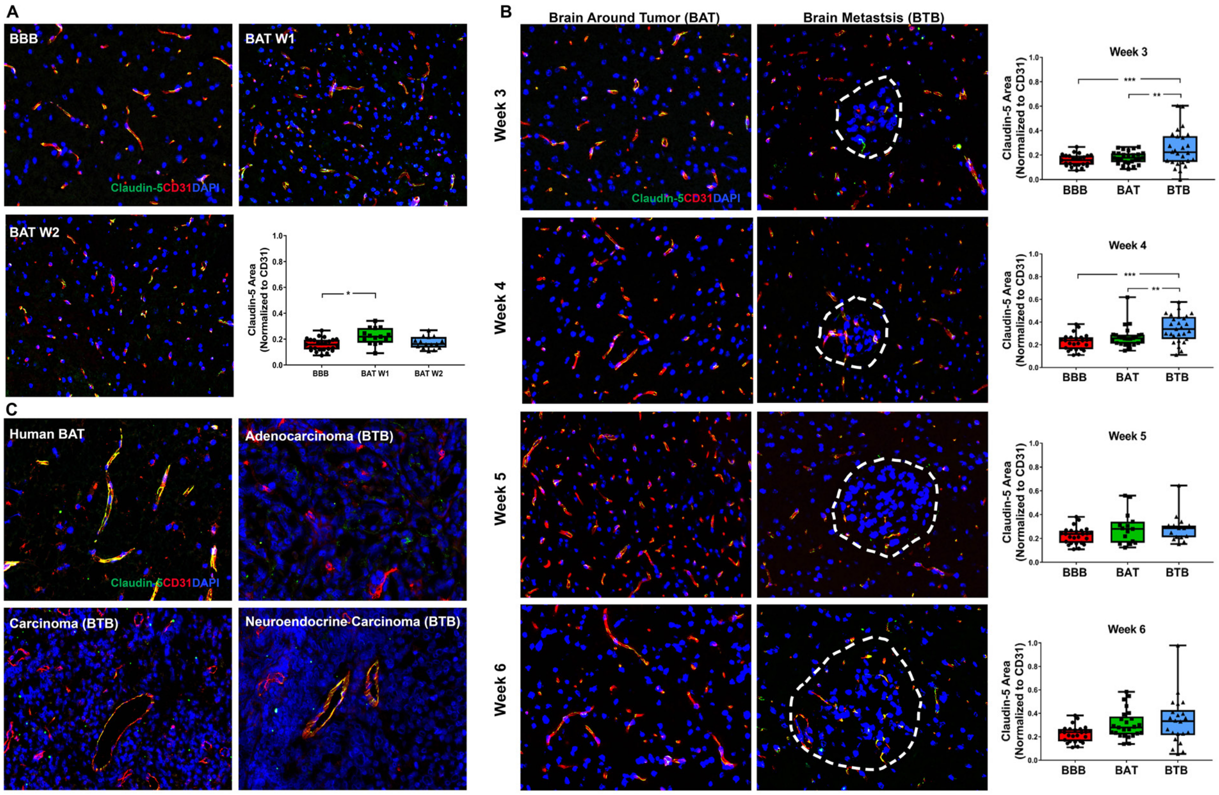

Supplementary Figure 4: Increased claudin-5 expression in brain metastases of NSCLC. Representative immunofluorescence microscopy images of claudin-5 (green) costained with CD31 (red) and DAPI (blue) in early-stage (**A**), mid- and late-stage (**B**) metastases in experimental NSCLC brain metastases and human adenocarcinoma, carcinoma and neuroendocrine carcinoma specimens (**C**). All images were acquired at 200X total magnification. Tumor margins are highlighted with a white dashed line. Within the box and whisker plot, the black line represents the mean of the data collected, box boundaries represent the 25th and 75th percentile, and error bars extend to the minimum and maximum data points. Each data point within the BAT and BBB groups represent a single image from a single animal. Each data point within the BTB group represents a single metastasis from a single animal. The level of significance was set at $p < 0.05$ ($^{*}p < 0.05$; $^{**}p < 0.01$; $^{***}p < 0.001$). (A) There was a significant increase in claudin-5 expression at one-week post-ICI ($n = 3$) compared to the BBB ($n = 5$). (B) In mid-stage NSCLC brain metastases, there was a significant increase in claudin-5 expression in the BTB ($n = 5$) compared to the BAT ($n = 5$) and BBB ($n = 5$). (C) Claudin-5 expression was lost in human NSCLC brain metastases capillaries.

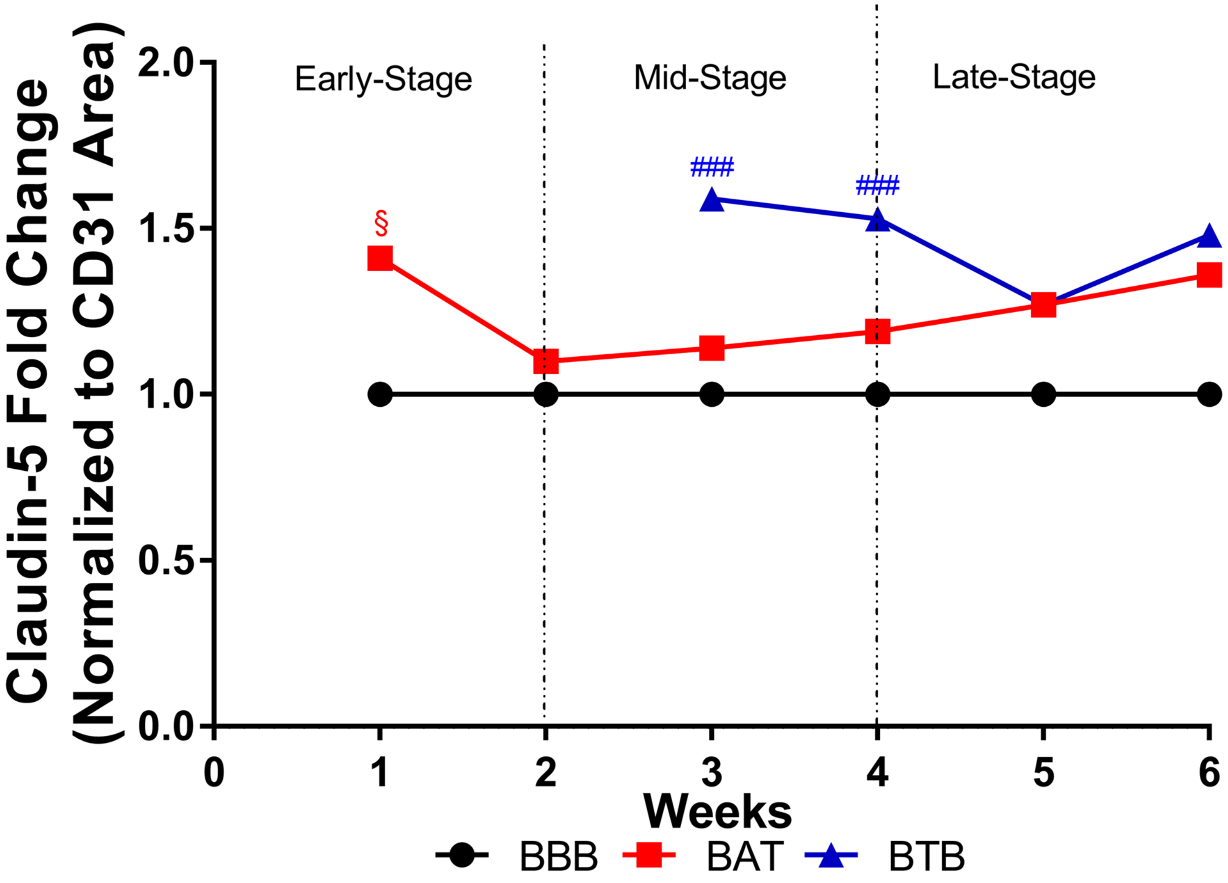

Supplementary Figure 5: Time-dependent quantitative analysis of claudin-5 expression within the BBB, BAT, and BTB in NSCLC brain metastases. There was a gradual increase in claudin-5 expression in the BAT over time. The line graph demonstrates quantitative analysis of claudin-5 in early stage (1–2 weeks), mid-stage (3–4 weeks) and late stage (5–6 weeks) metastases. The BBB is held constant at 1.0, and each data point represents the overall fold-change within the BTB and BAT. The level of significance was set at $p < 0.05$ ($^{\#,\,\S}p < 0.05$; $^{\#\#,\,\S\S}p < 0.01$; $^{\#\#\#,\,\S\S\S}p < 0.001$).

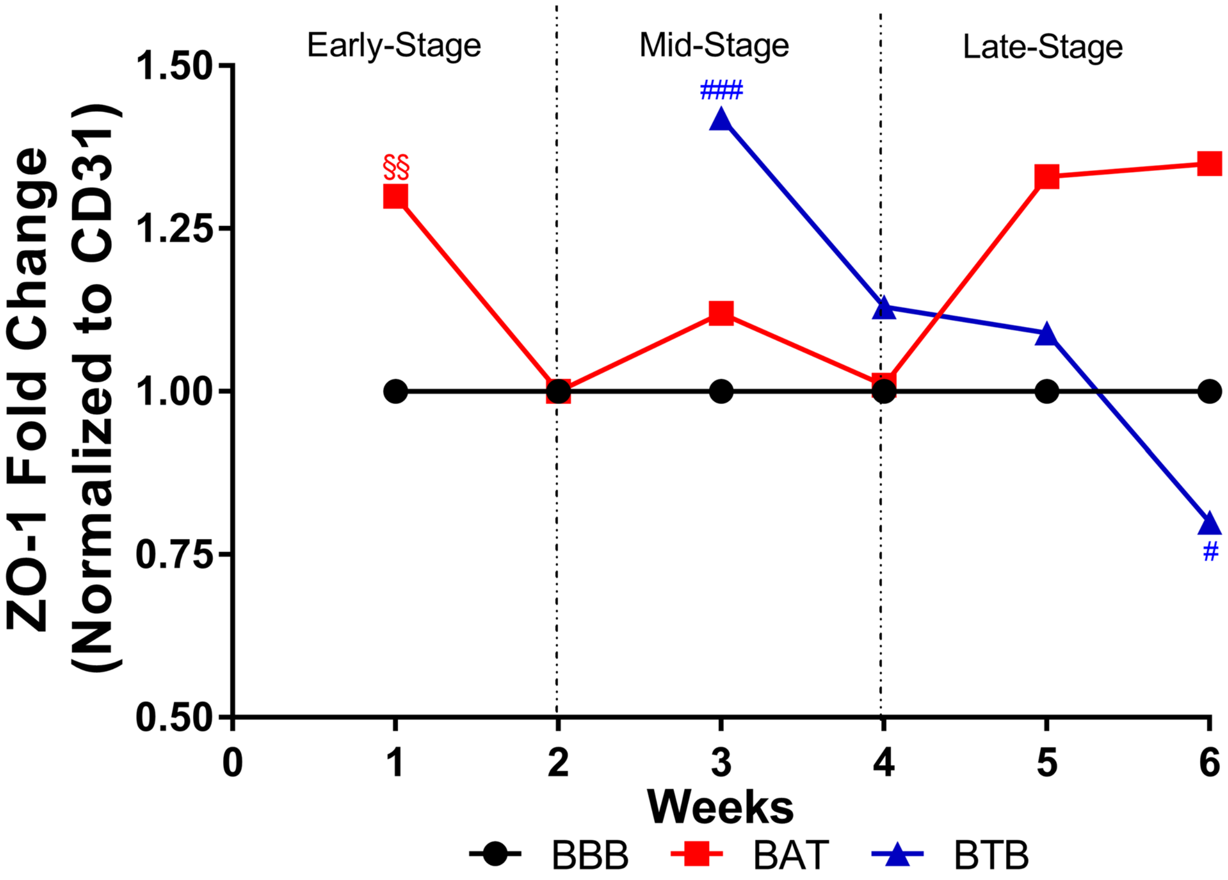

Supplementary Figure 6: Time-dependent quantitative analysis of ZO-1 expression within the BBB, BAT, and BTB in NSCLC brain metastases. The line graph demonstrates quantitative analysis of ZO-1 in early stage (1–2 weeks), mid-stage (3–4 weeks) and late stage (5–6 weeks) metastases. The BBB is held constant at 1.0, and each data point represents the overall fold-change within the BTB and BAT. The level of significance was set at $p < 0.05$ ($^{\#,\S}p < 0.05$; $^{\#\#,\S\S}p < 0.01$; $^{\#\#\#,\S\S\S}p < 0.001$).

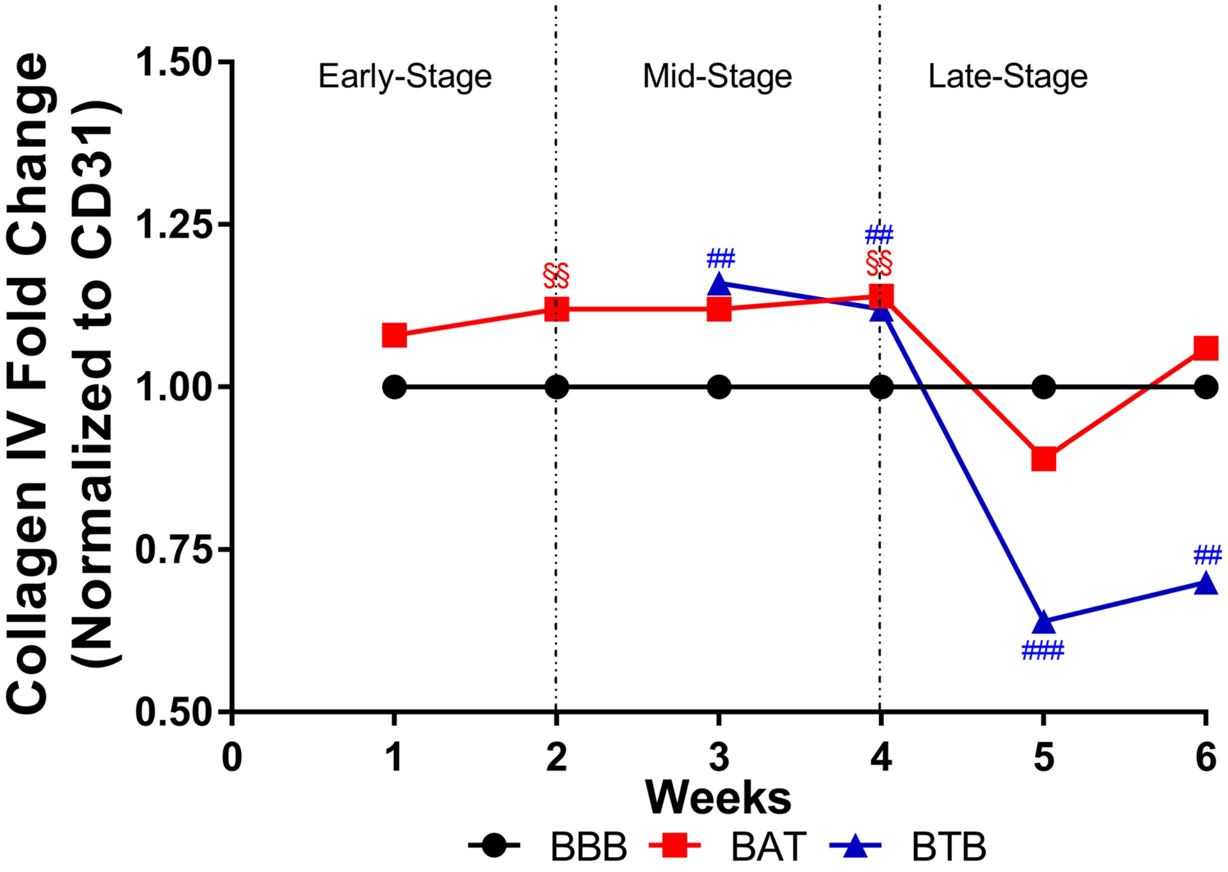

Supplementary Figure 7: Time-dependent quantitative analysis of Collagen IV expression within the BBB, BAT, and BTB in NSCLC brain metastases. The line graph demonstrates quantitative analysis of Collagen IV in early stage (1–2 weeks), mid-stage (3–4 weeks) and late stage (5–6 weeks) metastases. The BBB is held constant at 1.0, and each data point represents the overall fold-change within the BTB and BAT. The level of significance was set at $p < 0.05$ ($^{\#, \S}p < 0.05$; $^{\#\#, \S\S}p < 0.01$; $^{\#\#\#, \S\S\S}p < 0.001$).

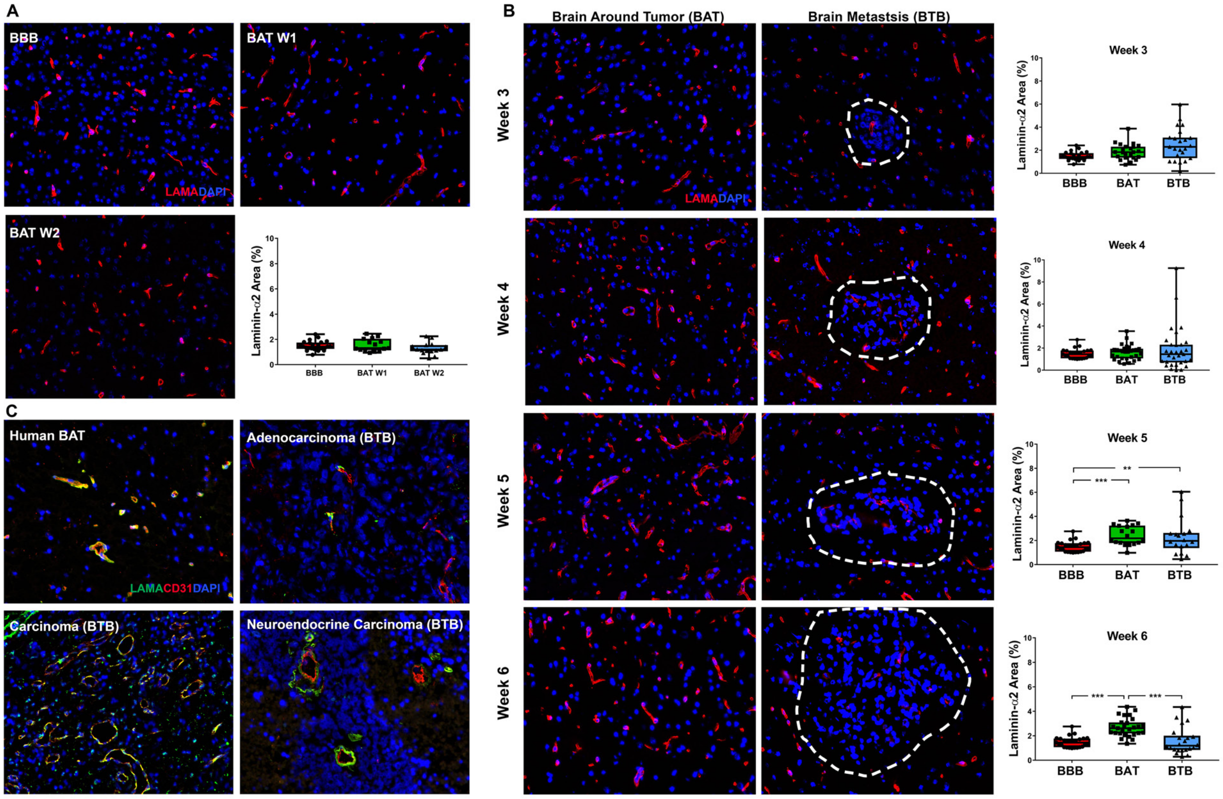

Supplementary Figure 8: Increased Laminin-α2 expression in BAT throughout the late-stage experimental model NSCLC brain metastases. Representative immunofluorescence microscopy images of Laminin-α2 (red) costained with and DAPI (blue) at early-stage (**A**), mid- and late-stage (**B**) metastases in experimental NSCLC brain metastases. In human adenocarcinoma, carcinoma and neuroendocrine carcinoma specimens, Laminin-α2 (green) was costained with CD31 (red) and DAPI (blue) (**C**). All images were acquired at 200X total magnification. Tumor margins are highlighted with a white dashed line. Within the box and whisker plot, the black line represents the mean of the data collected, box boundaries represent the 25th and 75th percentile, and error bars extend to the minimum and maximum data points. Each data point within the BAT and BBB groups represent a single image from a single animal. Each data point within the BTB group represents a single metastasis from a single animal. The level of significance was set at $p < 0.05$ ($^{*}p < 0.05$; $^{**}p < 0.01$; $^{***}p < 0.001$). (A) At the early-stage metastases ($n = 3$), there was no significant change in LAMA2 protein expression compared to the BBB ($n = 5$). (B) At the mid-stage metastasis, there was a significant increase in LAMA2 expression in the BTB ($n = 5$) compared to the BAT ($n = 5$) and BBB ($n = 5$). LAMA2 expression was increased in BAT (week 5 $n = 3$, week 6 $n = 5$) compared to the BBB ($n = 5$) at the late-stage metastases. (C) In human NSCLC specimens, there was a loss of LAMA2 expression.

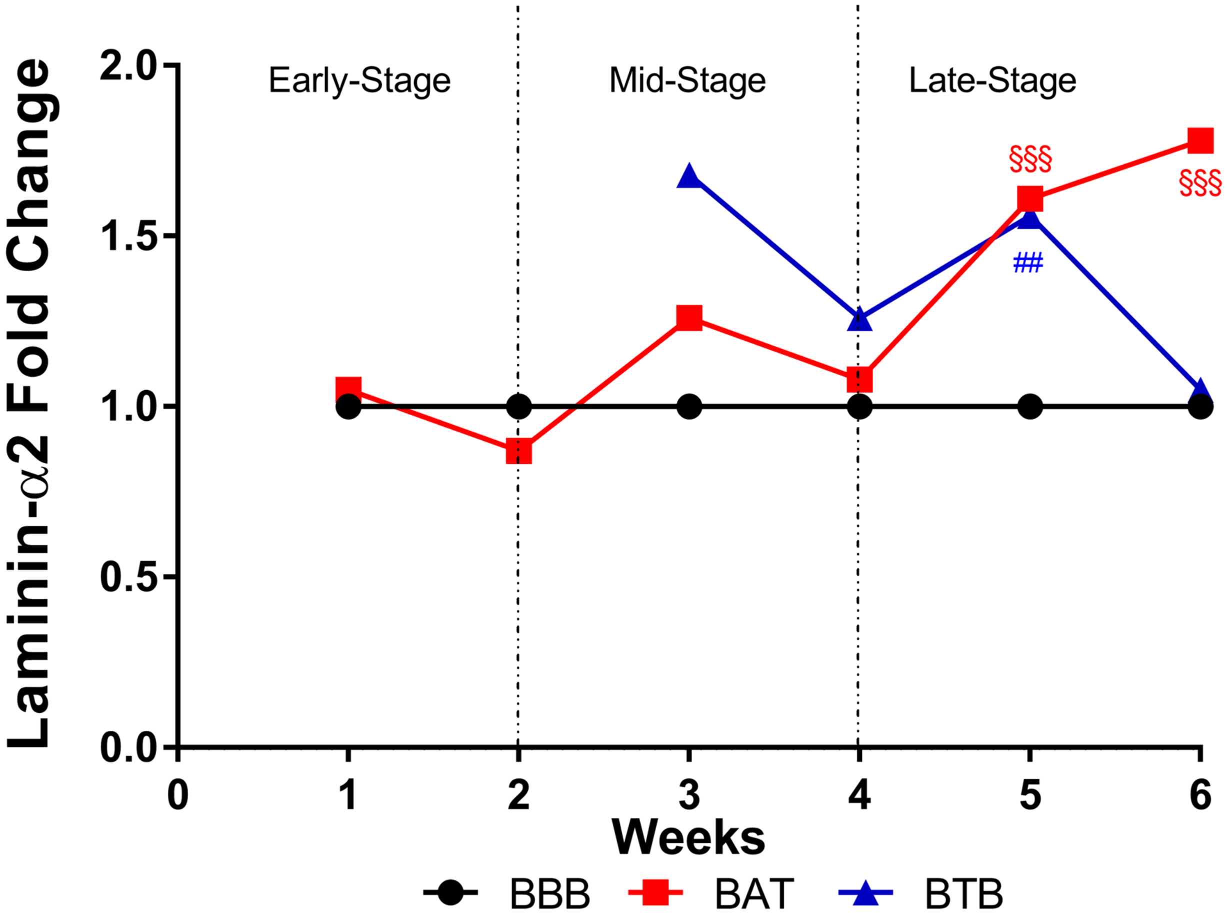

Supplementary Figure 9: Time-dependent quantitative analysis of Laminin-α2 expression within the BBB, BAT, and BTB in NSCLC brain metastases. There was a significant increase in LAMA2 expression within the BAT and BTB over time. The line graph demonstrates quantitative analysis of LAMA2 in early stage (1–2 weeks), mid-stage (3–4 weeks) and late stage (5–6 weeks) metastases. The BBB is held constant at 1.0, and each data point represents the overall fold-change within the BTB and BAT. The level of significance was set at $p < 0.05$ ($^{\#,\S}p < 0.05$; $^{\#\#,\S\S}p < 0.01$; $^{\#\#\#,\S\S\S}p < 0.001$).

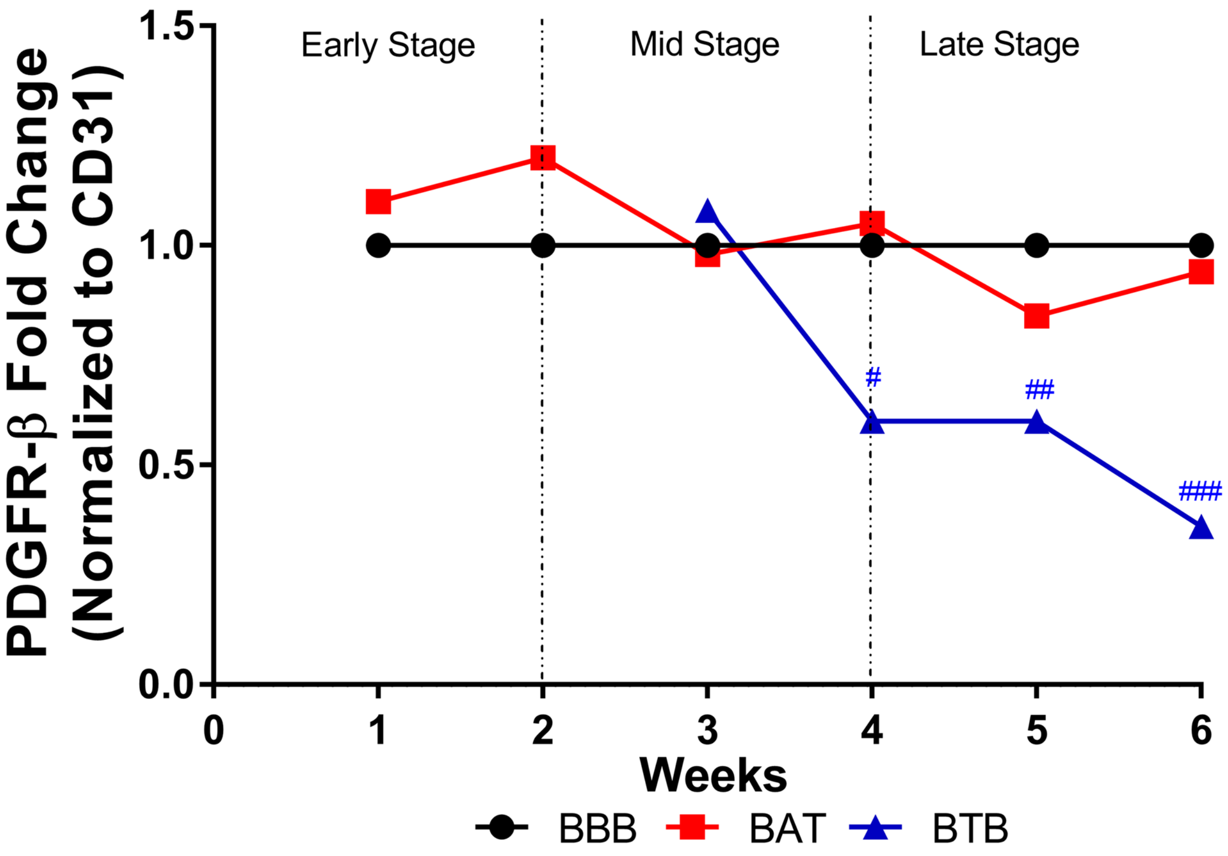

Supplementary Figure 10: Time-dependent quantitative analysis of PDGFR-β expression within the BBB, BAT, and BTB in NSCLC brain metastases. There was a gradual decrease in PDGFR-β expression in the BTB over time. The line graph demonstrates quantitative analysis of PDGFR-β in early stage (1–2 weeks), mid-stage (3–4 weeks) and late stage (5–6 weeks) metastases. The BBB is held constant at 1.0, and each data point represents the overall fold-change within the BTB and BAT. The level of significance was set at $p < 0.05$ ($^{\#, \S}p < 0.05$; $^{\#\#, \S\S}p < 0.01$; $^{\#\#\#, \S\S\S}p < 0.001$).

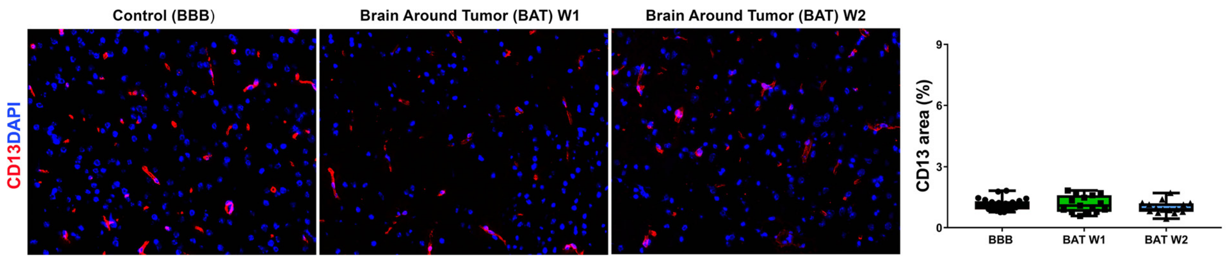

Supplementary Figure 11: CD13 expression within the early-stage of experimental NSCLC brain metastases. Representative immunofluorescence microscopy images of CD13 (red) costained with DAPI (blue) at early-stage metastases in experimental NSCLC brain metastases. All images were acquired at 200X total magnification. Within the box and whisker plot, the black line represents the mean of the data collected, box boundaries represent the 25th and 75th percentile, and error bars extend to the minimum and maximum data points. Each data point within the BAT and BBB groups represent a single image from a single animal. Each data point within the BTB group represents a single metastasis from a single animal. The level of significance was set at $p < 0.05$. At the early-stage metastases (n = 3), there was no significant change in CD13 protein expression compared to the BBB (n = 5).

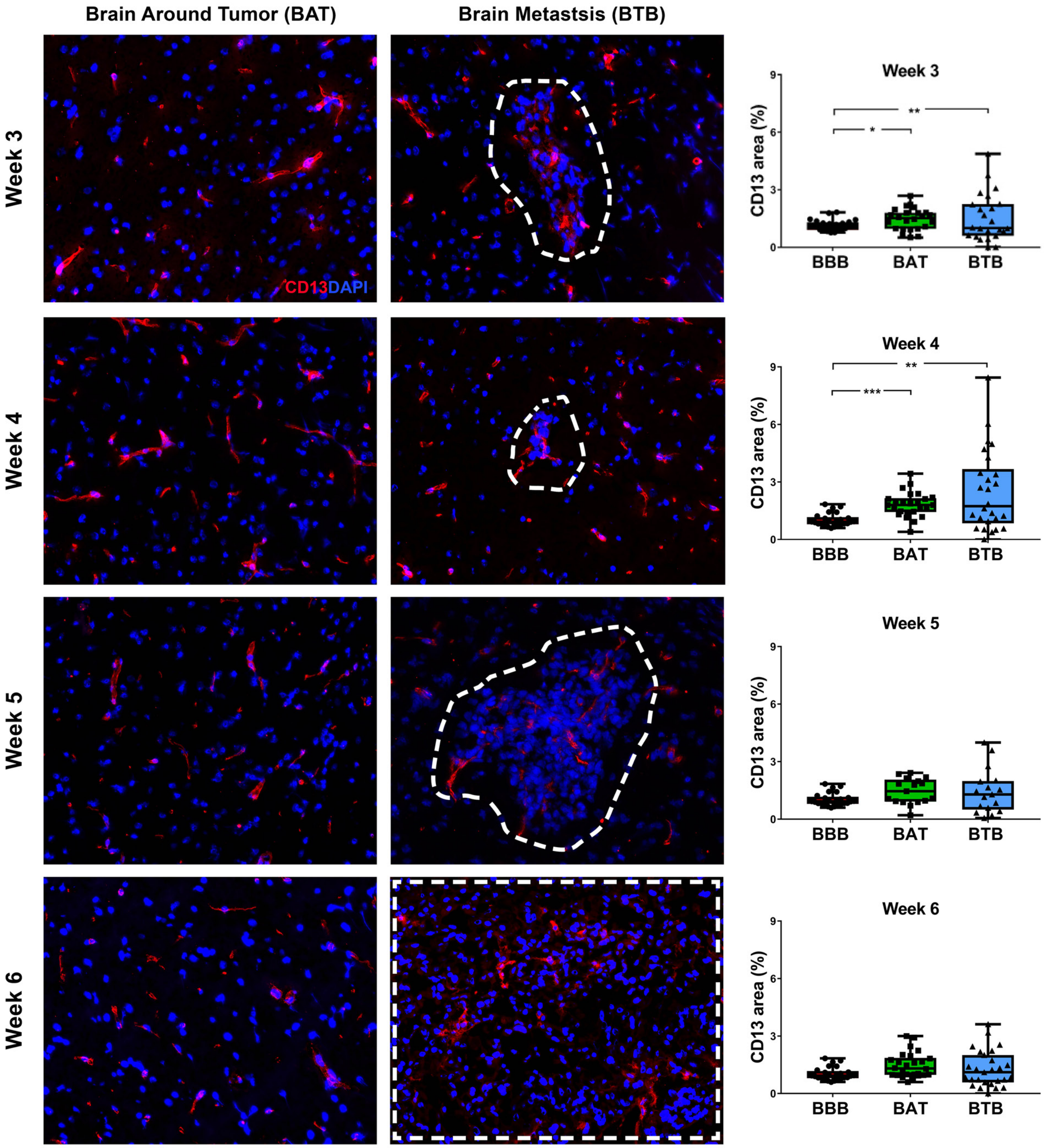

Supplementary Figure 12: CD13 expression within mid- and late-stage metastasis of experimental NSCLC brain metastases. Representative immunofluorescence microscopy images of CD13 (red) costained with and DAPI (blue) at mid- and late-stage metastases in experimental NSCLC brain metastases. All images were acquired at 200X total magnification. Tumor margins are highlighted with a white dashed line. Within the box and whisker plot, the black line represents the mean of the data collected, box boundaries represent the 25th and 75th percentile, and error bars extend to the minimum and maximum data points. Each data point within the BAT and BBB groups represent a single image from a single animal. Each data point within the BTB group represents a single metastasis from a single animal. The level of significance was set at $p < 0.05$ ($^{*}p < 0.05$; $^{**}p < 0.01$; $^{***}p < 0.001$). At the mid-stage metastasis, there was a significant increase in CD13 expression in the BTB ($n = 5$) and BAT ($n = 5$) compared to the BBB ($n = 5$). However, CD13 expression remained same at the late-stage metastases (week 5 $n = 3$, week 6 $n = 5$) compared to the BBB ($n = 5$).

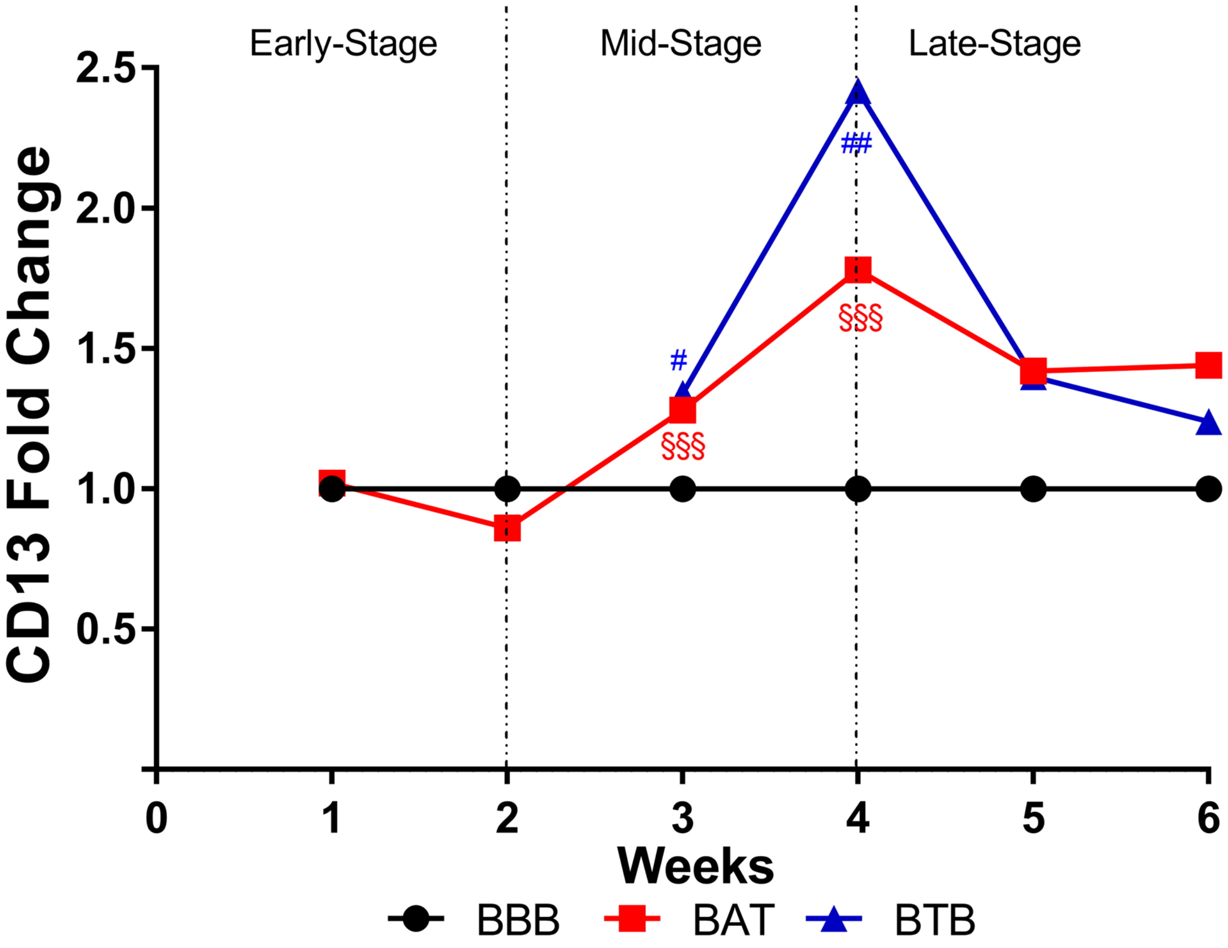

Supplementary Figure 13: Time-dependent quantitative analysis of CD13 expression within the BBB, BAT, and BTB in NSCLC brain metastases. There was a significant increase in CD13 expression in the BAT and BTB over time. The line graph demonstrates quantitative analysis of CD13 in early stage (1–2 weeks), mid-stage (3–4 weeks) and late stage (5–6 weeks) metastases. The BBB is held constant at 1.0, and each data point represents the overall fold-change within the BTB and BAT. The level of significance was set at $p < 0.05$ ($^{\#, \S}p < 0.05$; $^{\#\#, \S\S}p < 0.01$; $^{\#\#\#, \S\S\S}p < 0.001$).

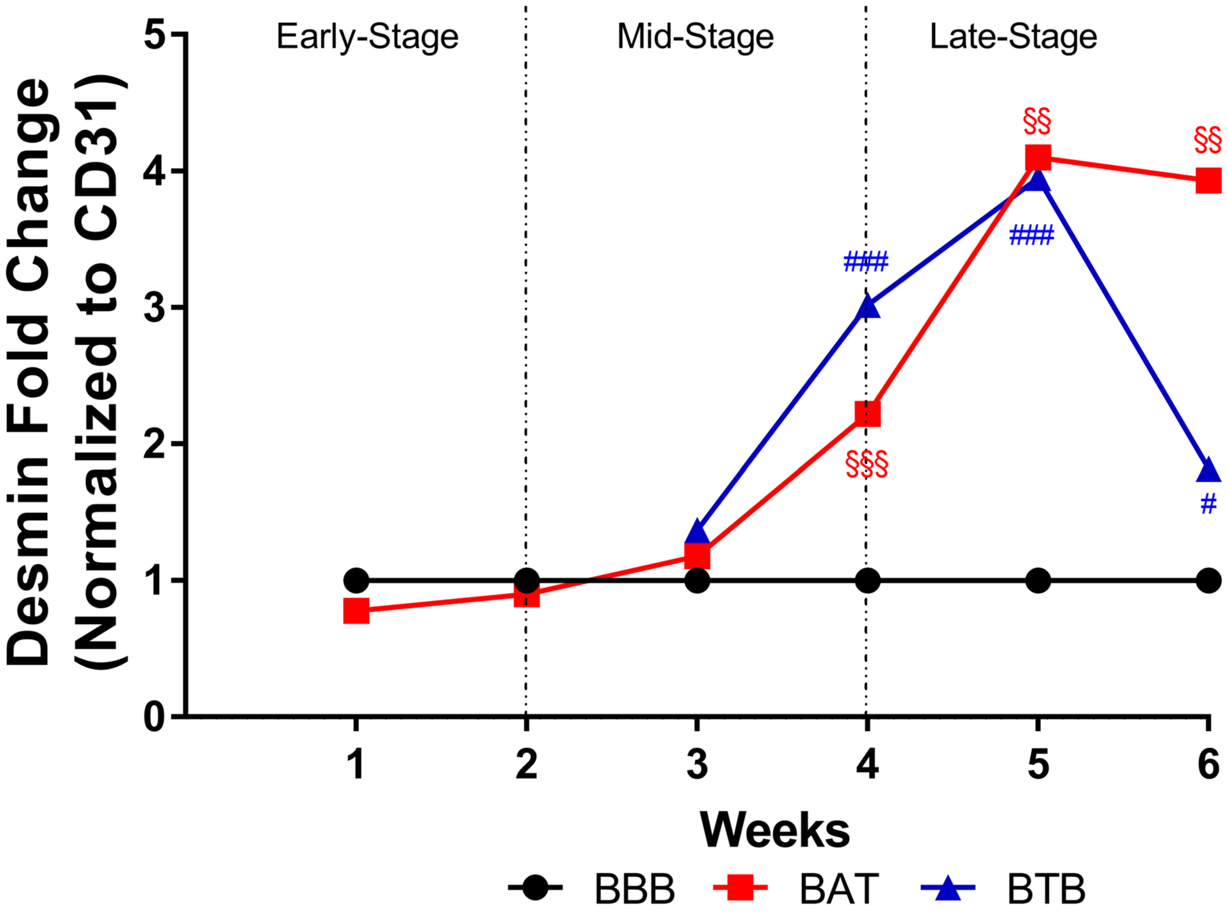

Supplementary Figure 14: Time-dependent quantitative analysis of desmin expression within the BBB, BAT, and BTB in NSCLC brain metastases. There was a significant increase in desmin expression in the BAT and BTB over time. The line graph demonstrates quantitative analysis of desmin in early stage (1–2 weeks), mid-stage (3–4 weeks) and late stage (5–6 weeks) metastases. The BBB is held constant at 1.0, and each data point represents the overall fold-change within the BTB and BAT. The level of significance was set at $p < 0.05$ ($^{\#,\S}p < 0.05$; $^{\#\#,\S\S}p < 0.01$; $^{\#\#\#,\S\S\S}p < 0.001$).

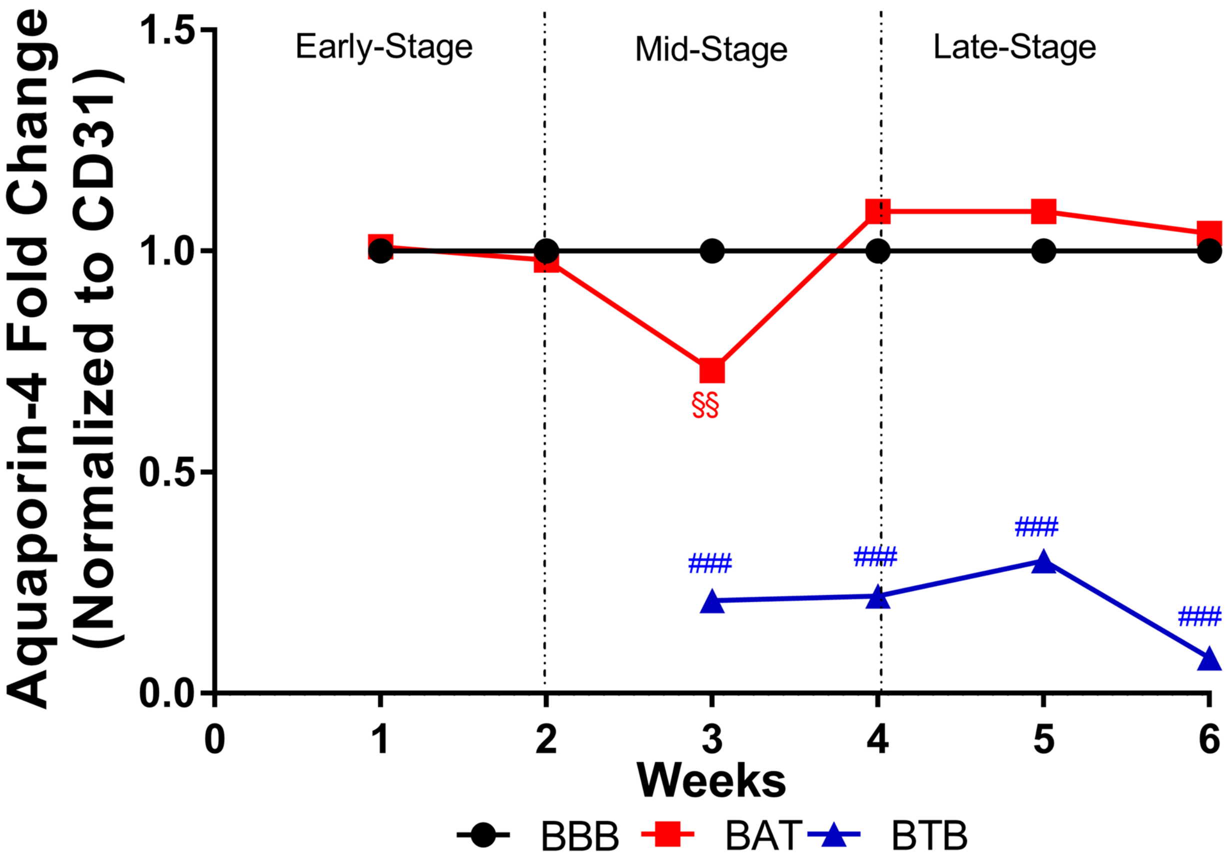

Supplementary Figure 15: Time-dependent quantitative analysis of aquaporin-4 expression within the BBB, BAT, and BTB in NSCLC brain metastases. There was a significant and dramatic loss of AQP4 expression in the BTB over time. The line graph demonstrates quantitative analysis of AQP4 in early stage (1–2 weeks), mid-stage (3–4 weeks) and late stage (5–6 weeks) metastases. The BBB is held constant at 1.0, and each data point represents the overall fold-change within the BTB and BAT. The level of significance was set at $p < 0.05$ ($^{\#,\ \S}p < 0.05$; $^{\#\#,\ \S\S}p < 0.01$; $^{\#\#\#,\ \S\S\S}p < 0.001$).

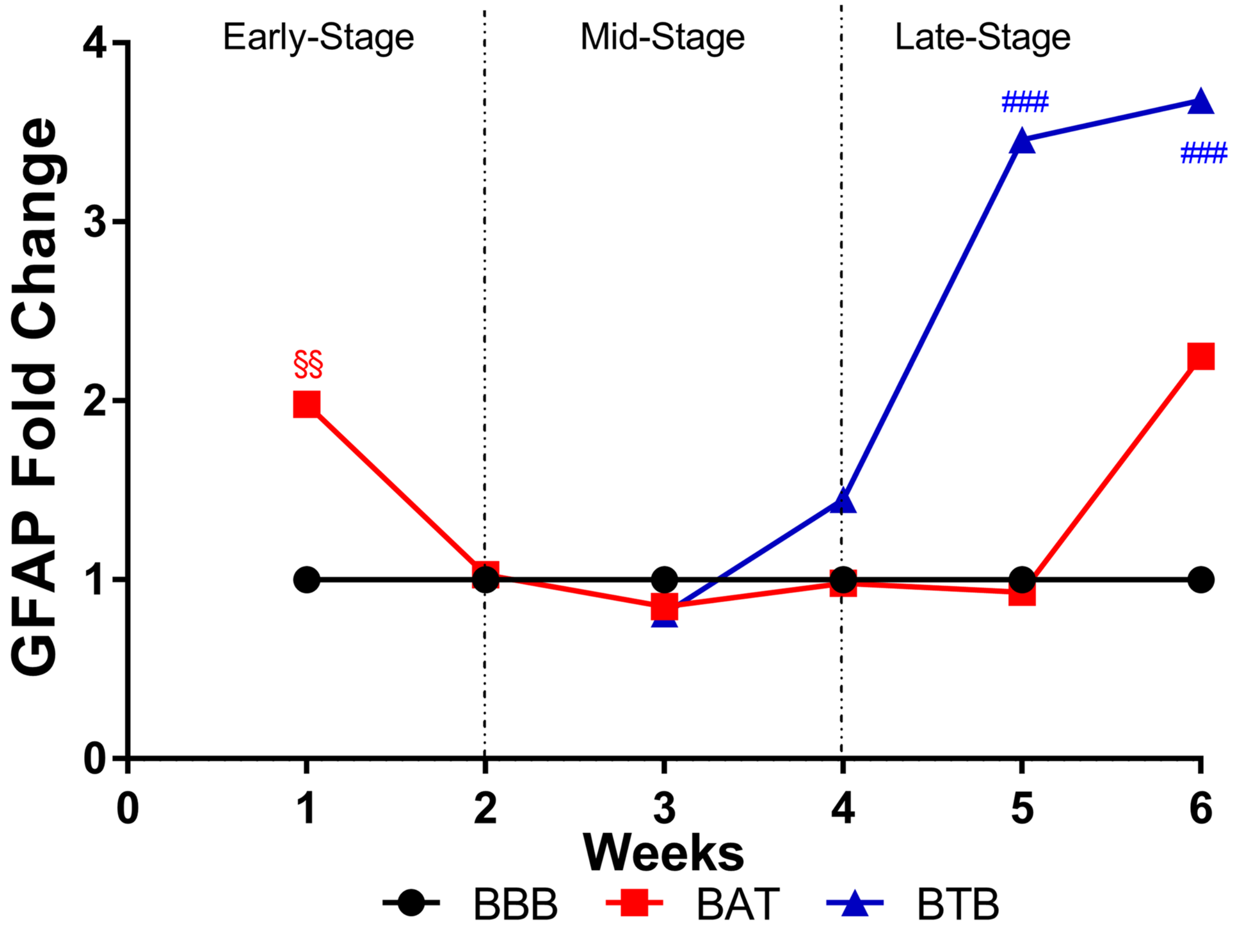

Supplementary Figure 16: Time-dependent quantitative analysis of GFAP expression within the BBB, BAT, and BTB in NSCLC brain metastases. There was a significant increase in GFAP expression in the BAT and BTB over time. The line graph demonstrates quantitative analysis of GFAP in early stage (1–2 weeks), mid-stage (3–4 weeks) and late stage (5–6 weeks) metastases. The BBB is held constant at 1.0, and each data point represents the overall fold-change within the BTB and BAT. The level of significance was set at $p < 0.05$ ($^{\#,\S}p < 0.05$; $^{\#\#,\S\S}p < 0.01$; $^{\#\#\#,\S\S\S}p < 0.001$).
